# The mitochondrial genome of *Acrobeloides varius* (Cephalobomorpha) confirms non-monophyly of Tylenchina (Nematoda)

**DOI:** 10.7717/peerj.9108

**Published:** 2020-05-13

**Authors:** Taeho Kim, Yucheol Lee, Hyun-Jong Kil, Joong-Ki Park

**Affiliations:** 1 Division of EcoScience, Ewha Womans University, Seoul, Republic of Korea; 2 Animal Resources Division, National Institute of Biological Resources, Incheon, Republic of Korea

**Keywords:** *Acrobeloides varius*, Cephalobomorpha, Tylenchina, Mitochondrial genome, Phylogeny

## Abstract

The infraorder Cephalobomorpha is a diverse and ecologically important nematode group found in almost all terrestrial environments. In a recent nematode classification system based on SSU rDNA, Cephalobomorpha was classified within the suborder Tylenchina with Panagrolaimomorpha, Tylenchomorpha and Drilonematomorpha. However, phylogenetic relationships among species within Tylenchina are not always consistent, and the phylogenetic position of Cephalobomorpha is still uncertain. In this study, in order to examine phylogenetic relationships of Cephalobomorpha with other nematode groups, we determined the complete mitochondrial genome sequence of *Acrobeloides varius*, the first sequenced representative of Cephalobomorpha, and used this sequence for phylogenetic analyses along with 101 other nematode species. Phylogenetic analyses using amino acid and nucleotide sequence data of 12 protein-coding genes strongly support a sister relationship between the two cephalobomorpha species *A. varius* and *Acrobeles complexus* (represented by a partial mt genome sequence)*.* In this mitochondrial genome phylogeny, Cephalobomorpha was sister to all chromadorean species (excluding *Plectus acuminatus* of Plectida) and separated from Panagrolaimomorpha and Tylenchomorpha, rendering Tylenchina non-monophyletic. Mitochondrial gene order among Tylenchina species is not conserved, and gene clusters shared between *A. varius* and *A*.* complexus* are very limited. Results from phylogenetic analysis and gene order comparison confirms Tylenchina is not monophyletic. To better understand phylogenetic relationships among Tylenchina members, additional mitochondrial genome information is needed from underrepresented taxa representing Panagrolaimomorpha and Cephalobomorpha.

## Introduction

The suborder Tylenchina *sensu* De Ley & Blaxter (2002) represents one of the most morphologically and ecologically diverse arrays of nematodes and contains many different trophic types: microbivores, fungivores, and plant- and animal-parasitic forms (Baldwin, Ragsdale & Bumbarger, 2004; Bert et al., 2008; Siddiqi, 1980). In the SSU rDNA-based classification system for the phylum Nematoda, the suborder Tylenchina was initially classified to include four infraorders (Cephalobomorpha, Drilonematomorpha, Panagrolaimomorpha and Tylenchomorpha) (De Ley & Blaxter, 2002; De Ley & Blaxter, 2004).

Cephalobomorphs, one of the infraordinal groups in Tylenchina, are the dominant fauna together with other nematodes in nutrient-poor soil such as deserts and dry Antarctic valleys and may play important roles in shaping ecological systems there (Andriuzzi et al., 2018; De Tomasel et al., 2013; Freckman & Mankau, 1986; Freckman & Virginia, 1997; Lee et al., 2019; Sylvain et al., 2014). Additionally, this group constitutes a microbivorous or saprophagous clade that is found in diverse terrestrial environments including tropical rainforests and hot deserts (Nadler et al., 2006; Timm, 1971; Waceke et al., 2005). Historically, relationships between cephalobomorphs and other chromadorean nematodes inferred using morphology have produced considerably different hypotheses. On the basis of similarities in stoma and oesophagus features, cephalobomorphs were considered closely related to rhabditomorphs in many previous nematode taxonomies (Chitwood & Chitwood, 1977; Maggenti, 1981; Poinar, 1983). On the other hand, other authors proposed that cephalobomorphs had a close relationship with parasitic, fungivore and plant parasitic tylenchs (Siddiqi, 1980; Siddiqi, 2000), the entomopathogenic steinernematids (Poinar, 1993) and/or annelid-parasitic drilonematids (Coomans & Goodey, 1965; De Ley & Coomans, 1990; Spiridonov, Ivanova & Wilson, 2005).

In the first phylum-wide molecular phylogeny of nematodes using nuclear small subunit ribosomal DNA (SSU rDNA) sequences, cephalobs were placed in Clade IV with panagrolaimids, strongyloids, steinernematids and tylenchs (Blaxter et al., 1998). In a further revised nematode classification system (De Ley & Blaxter, 2004) following the SSU phylogeny, cephalobs were treated as the infraorder Cephalobomorpha and placed in the suborder Tylenchina, along with Drilonematomorpha, Panagrolaimomorpha and Tylenchomorpha. These relationships were supported in subsequent phylogenetic analyses using SSU rDNA data with more extensive taxon sampling (Holterman et al., 2006; Van Megen et al., 2009). However, relationships among subordinate taxa within Tylenchina were not always consistent, depending on the size of the datasets analyzed (Holterman et al., 2006; Van Megen et al., 2009). Thus, alternative genetic markers are needed to elucidate the relationships that SSU rDNA data are not sufficient to resolve.

Complete mitochondrial genomes have been used for phylogenetic analyses of diverse animal groups and are a powerful molecular marker for resolving not only deep phylogeny but also relationships between groups with relatively recent common ancestry (Hegedusova et al., 2014; Hu & Gasser, 2006; Littlewood et al., 2006; Liu et al., 2013; Park et al., 2007; Waeschenbach et al., 2006). In nematode phylogenetic analyses based on complete mitochondrial genome sequences, inferred relationships have been mostly similar to SSU rRNA or morphology-based phylogenies. However, a mitochondrial genome approach has provided alternative hypotheses that differed from nuclear gene phylogeny in some cases, such as non-monophyly of clade III (*sensu* Blaxter et al., 1998), non-monophyly of Tylenchomorpha, and strong support for a clade consisting of the infraorders Rhabditomorpha, Diplogasteromorpha, Ascaridomorpha and Rhigonematomorpha (Kim et al., 2017; Kim et al., 2014).

Although available mt genome information for nematodes has been increasing in recent years, some nematode groups, including free-living microbivores and insect-associated taxa, are still underrepresented. In fact, among the 200 currently available nematode mt genomes (in GenBank as of December 2019), no representatives from Cephalobomorpha are yet available: only a partial fragment (contains *cox1*-*cox3*, *nad2*-*nad6*, *nad4l* and 15 tRNA genes) of the *Acrobeles complexus* mt genome (see Kim et al., 2015 for details). In this study, we determined the complete mitochondrial genome sequence of *Acrobeloides varius*, the first full representative of the infraorder Cephalobomorpha, compared its genome structure and organization to other nematodes, and used it to infer phylogenetic relationships among chromadorean nematodes.

## Material and Methods

### Sampling, culturing and identification of specimens

Specimens were obtained from soil collected from agricultural (rice) farmland in Hapcheon-gun, Gyeongsangnam Province, South Korea. Species identification was performed by a careful examination of morphological characters and morphometric analysis following the protocols of a recently published study (Kim, Kim & Park, 2017). For culturing, one individual nematode was transferred onto a soil agar plate (25 mg/ml autoclaved soil, 5 ug/ml cholesterol and 1% agar) at room temperature. Specimens were subsequently selected from the culture plate; roughly half of all selected specimens were used for species identification (see Kim, Kim & Park, 2017); the other half were preserved in absolute ethanol and stored at −80 °C until total genomic DNA was extracted. To reconfirm species identification, *cox1* sequences from each individual were compared with *cox1* sequences of the specimens published in Kim, Kim & Park (2017).

### Molecular techniques

Total genomic DNA was extracted from about 10 individuals using a lysis buffer containing 0.2 M NaCl, 0.2 M Tris-HCL (pH 8.0), 1% (v/v) β-mercaptoethanol and 800 µg/ml proteinase-K (Holterman et al., 2006). Four partial fragments were initially amplified by polymerase chain reaction (PCR) for four different genes (*cox1*, *cob*, *rrnL* and *nad5*) using nematode-specific primer sets (Cepha_CO1_F/Cepha_CO1_R for *cox1*, Chroma_Cob_F/Chroma_Cob_R for *cob*, Nema_16S_F/Nema_16S_R for *rrnL* and Nema_ND5_F/Nema_ND5_R for *nad5*) designed from conserved regions of other nematode mitochondrial gene sequences available on GenBank (Table 1). PCR amplification was carried out in total 50 µL-volume reactions consisting of 0.2 mM dNTP mixture, 1X Ex Taq buffer containing MgCl_2_, 10 pmol primers (for both forward and reverse primers), 1.25 U Taq polymerase (TaKaRa Ex Taq), and 2 µL template with the following amplification conditions: one cycle of initial denaturing at 95 °C for 1 min, followed by 35 cycles of denaturation at 95 °C for 30 s, annealing at 47 °C for 30 s, extension at 72 °C for 1 min, and one cycle of final extension at 72 °C for 10 min. The nucleotide sequences obtained from respective partial DNA fragments were then used to design species-specific primer sets (Table 1) for long PCR amplification. PCR reactions were carried out in 50 µL total volumes consisting of 0.4 mM dNTP mixture, 1X LA Taq buffer, 2.5 mM MgCl_2_, 10 pmol primers (both forward and reverse primers), 2.5 U Taq polymerase (TaKaRa LA Taq), and 2 µL template with the following amplification conditions: one cycle of initial denaturing at 95 °C for 1 min, followed by 35 cycles of denaturation at 95 ° C for 30 s, annealing and extension at 55 °C to 70  °C for 2 min to 10 min, and one cycle of final extension at 68 °C for 10 min. The amplified PCR products were isolated on 1% agarose gels using a QIAquick Gel Extraction Kit (QIAGEN Co.) following standard protocols. Two long-PCR products (Acva_16S_F/Acva_ND5_R and Acva_ND5_F/Acva_Cob_R) were used directly for primer walking sequencing*.* The other long-PCR products (Acva_CO1_F/Acva_16S_R and Acva_Cob_F/Acva_CO1_R) were ligated into a pCR-XL-TOPO vector using a TOPO XL PCR Cloning Kit (Invitrogen Co.) and then transformed into One Shot TOP10 Electrocompetent *E. coli* using electroporation (1.8 kV). Plasmid DNAs were extracted from competent cells using a QIAprep Spin Miniprep Kit (QIAGEN Co.) following standard protocols. Sequences of the PCR-amplified and/or cloned DNA fragments were determined using a Big Dye Terminator Cycle-Sequencing kit and ABI PRISM 3730XL Analyzer (Applied Biosystem Co.). A complete strand of the entire mitochondrial DNA sequence was then assembled by reconfirming the sequences of the overlapping regions of the long PCR fragments and partial fragments.

**Table 1:** Primers used to sequence the complete mitochondrial genome of *Acrobeloides varius*.

Primers	Sequence (5′→3′)	Estimated size of PCR products
Cepha_CO1_F	ATGATTTTTTTTATGGTGATGCC	700 bp
Cepha_CO1_R	ACTACAAAATATGTGTCATG	
Nema_16S_F	WWTAAATGGCAGYCTTAGCGTGA	450 bp
Nema_16S_R	TCTYMCRAYGAAYTAAACTAATATC	
Nema_ND5_F	GTTCATAGAAGTACTTTGGTKACTGCTG	500 bp
Nema_ND5_R	AAGACGMWAACWATAAMHAAAAGT	
Chroma_Cob_F	CARATRWSNTWTTGRGC	370 bp
Chroma_Cob_R	TAYCAYTCNGGNACAAYATG	
Acva_CO1_F	GAGCGCATCATATGTATGTAACTGG	6 kb
Acva_16S_R	CTACCCAAGGCGTCGTGTTCATCAC	
Acva _16S_F	CTTAGCGTGCGGACTTTAATGTAGC	2 kb
Acva _ND5_R	GAACCCACAGAATTTCACTTGCGTC	
Acva _ND5_F	CATGTGGGTTCTAATCAACAAAATTGACG	2.1 kb
Acva _Cob_R	ACACCTCTGTGAACGTAAAGCACAC	
Acva _Cob_F	GGTTATTAATGATAATCCTTATTATAGGTGC	8.5 kb
Acva _CO1_R	GATCTACAGAAGCTCCATAATGAC	

### Gene annotation and phylogenetic analyses

The 12 protein-coding genes (PCGs) and two ribosomal RNA genes were identified using the web-based automatic annotation programs DOGMA (http://dogma.ccbb.utexas.edu) (Wyman, Jansen & Boore, 2004) and MITOS (http://mitos.bioinf.uni-leipzig.de/index.py) (Bernt et al., 2013). The boundaries of each gene were confirmed by comparing nucleotide sequences with those from closely related nematodes. Putative secondary structures of 24 tRNAs were inferred using tRNAscan-SE Search Server v.1.21 (Lowe & Eddy, 1997), MITOS (Bernt et al., 2013) and DOGMA (Wyman, Jansen & Boore, 2004), and tRNA secondary structures and anticodon sequences were then checked manually.

For phylogenetic analysis, a representative nematode species was chosen from each genus for which a complete mitochondrial genome was available from GenBank (Table S1). Nucleotide and amino acid sequences of the 12 PCGs of 102 nematode species including *A. varius* and two arthropod species (outgroups) were concatenated for the analyses. In the nucleotide (NT) dataset, two datasets (3rd codon positions included and excluded were prepared for the analysis to examine whether tree topology is affected by nucleotide substitution saturation, which is most likely to occur in the third codon position. Multiple alignments of amino acid sequences of 12 PCGs were done individually using MAFFT with default options (Katoh & Standley, 2013). Nucleotide sequences of the 12 individual PCGs were also aligned with a scaffold of aligned amino acid sequences using RevTrans (Wernersson & Pedersen, 2003).

For phylogenetic analysis, individual amino acid and nucleotide alignments of 12 PCGs were both concatenated using Geneious Pro 10.2.3 (Biomatters Co.). The phylogenetic analyses utilizing each concatenated sequence dataset (amino acid (AA) sequences (4,532 AA residues) and nucleotide (NT) sequences with third codon position included (13,596 bp) or excluded (9,064 bp)) were performed using maximum likelihood (ML) with RAxML 8.2.10 (Stamatakis, 2014) and using Bayesian inference (BI) with MrBayes version 3.2.6 (Huelsenbeck & Ronquist, 2001); both programs were run through the CIPRES portal (Miller, Pfeiffer & Schwartz, 2010). The AA and NT sequence alignments were deposited in TreeBASE (https://www.treebase.org (ID number: 25588)). For phylogenetic analyses, the best-fit substitution model for each amino acid sequence dataset was estimated using the Akaike Information Criterion (AIC) as implemented in ProtTest 3.4.2 (Darriba et al., 2011) (Table S2). Substitution models for the two types of nucleotide sequence datasets (including versus excluding 3rd codon positions) were estimated separately for each gene using the AIC as implemented in jModelTest (Darriba et al., 2012). For the ML analysis, each of the 12 PCGs was treated as a separate partition and the best-fit substitution model (JTT, LG, MtArt or Vt) for the amino acid dataset was applied (Table S2). Similarly, for the ML analysis of the two nucleotide sequence datasets, each of the 12 PCGs was also treated as a separate, unlinked data partition; for nucleotide datasets RAxML uses the GTR GAMMA model for finding the optimal tree, with the shape of the gamma distribution estimated separately for each partition. Bootstrap maximum likelihood analysis was performed using the rapid bootstrapping option with 1000 iterations. For the BI analysis, the LG+I+G model of substitution (the closest fit model available for use in CIPRES) was used for the amino acid dataset, and the best-fit models for each of the 12 PCGs were used for the two nucleotide datasets (Table S2). Bayesian analysis was performed using four MCMC chains for 1 × 10^6^ generations, sampled every 100 generations. Bayesian posterior probability (BPP) values were estimated after discarding the initial 25 × 10^4^ generations as burn-in.

## Results and Discussion

### The mitochondrial genome of *A. varius*

**Figure 1:**
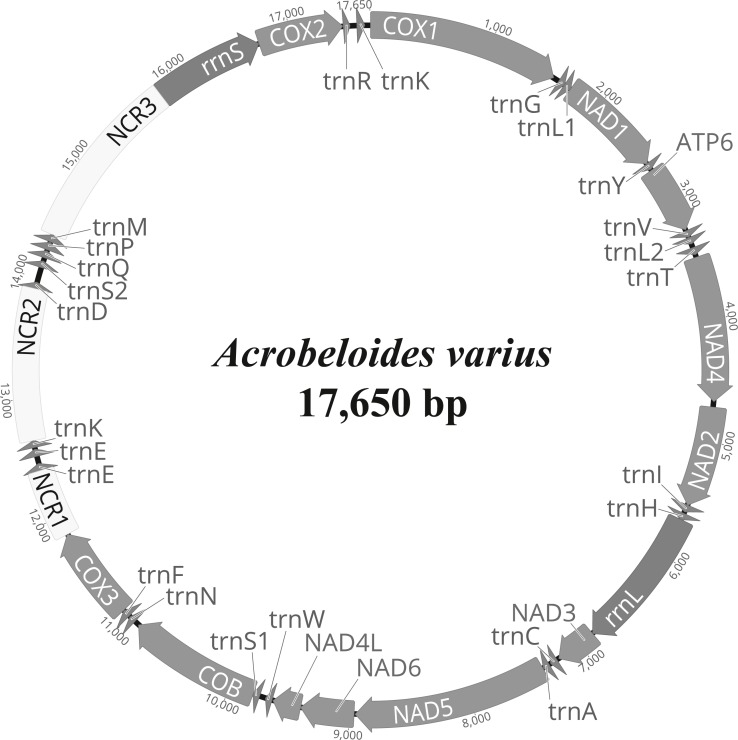
Representation of circular mitochondrial genome of *Acrobeloides varius*. All genes are encoded in the clockwise direction and 22 tRNA genes are marked by a single-letter amino acid code, based on the International Union of Pure and Applied Chemistry (IUPAC) code. The two leucine and two serine tRNA genes are labeled according to their individual anticodon sequence, as L1 (*trnL1*-uag), L2 (*trnL2*-uaa), S1 (*trnS1*-ucu), and S2 (*trnS2*-uga), respectively.

The complete mitochondrial genome of *A. varius* (GenBank accession no. MK559448) is composed of 17,650 bp and contains 12 PCGs (*atp6*, *cob*, *cox1*-*3*, *nad1*-*6* and *nad4L*), 22 tRNA genes and 2 rRNA genes (Fig. 1 and Table 2). The overall nucleotide composition of the *A. varius* mitochondrial DNA is 45.7% T, 8.8% C, 30.9% A, and 14.6% G, and the overall A+T content is 76.6% (Table S3). The overall A+T content is similar to that of other chromadorean nematodes, which ranges from 67.0% (*Ascaridia* sp. GHL-2013 (Liu et al., 2013) to 85.4% (*Radopholus similis* (Jacob et al., 2009)). In the 12 protein-coding mtDNA genes (PCGs), the most commonly used start codon is ATT, which is used for five genes (*atp6*, *cob*, *nad1*, *nad4* and *nad6*) (Table 2). Two genes (*cox2* and *nad3*) start with ATA, whereas *nad2* and *nad4l* are inferred to initiate with ATG and *cox1* with GTG, while *cox3* starts with TTG. The gene *nad5* uses TTT as the start codon. For termination codons, eight genes (*cob*, *cox2*, *cox3*, *nad2*, *nad3*, *nad4l*, *nad5* and *nad6*) appear to use TAA and four genes (*atp6*, *cox1*, *nad1* and *nad4*) use TAG. The codon usage of *A. varius* mtDNA is biased towards T-rich codons, similar to other nematode species. The four most frequently used codons are all T-rich (more than 2 Ts per triplet): TTT (10.6%), TTA (11.2%), ATT (7.5%) and TAT (5.4%), accounting for 34.7% of all PCGs (Table S4). In contrast, the frequency of C-rich codons (more than 2 Cs per triplet) is very low (2.9%).

**Table 2:** Mitochondrial genome organization of *Acrobeloides varius*.

Gene/ region	Position		Size		Codons		Intergenic sequence
	Start	Finish	No. of nt^a^	No. of aa^a^	Initiation	Termination	
*cox1*	1	1,575	1,575	524	GTG	TAG	48
*trnG*	1,624	1,680	57				0
*trnL1*	1,681	1,738	58				2
*nad1*	1,741	2,613	873	290	ATT	TAG	−1
*trnY*	2,613	2,668	56				9
*atp6*	2,678	3,238	561	186	ATT	TAG	11
*trnV*	3,250	3,305	56				3
*trnL2*	3,309	3,365	57				32
*trnT*	3,398	34,58	61				22
*nad4*	3,481	4,671	1,191	396	ATT	TAG	49
*nad2*	4,721	5,545	825	274	ATG	TAA	0
*trnI*	5,546	5,608	63				13
*trnH*	5,622	5,676	55				0
*rrnL*	5,677	6,859	1,183				0
*nad3*	6,860	7,204	345	114	ATA	TAA	18
*trnC*	7,223	7,280	58				17
*trnA*	7,298	7,353	56				4
*nad5*	7,358	8,950	1,593	530	TTT	TAA	12
*nad6*	8,963	9,400	438	145	ATT	TAA	−1
*nad4l*	9,400	9,633	234	77	ATG	TAA	7
*trnW*	9,641	9,699	59				47
*trnS1*	9,747	9,804	58				1
*cob*	9,806	10,900	1,095	364	ATT	TAA	40
*trnN*	10,941	10,997	57				10
*trnF*	11,008	11,067	60				3
*cox3*	11,071	11,844	774	257	TTG	TAA	0
*NCR1*	11,845	12,408	564				0
*trnE*	12,409	12,466	58				61
*trnE*	12,528	12,589	62				12
*trnK*	12,602	12,658	57				0
*NCR2*	12,659	13,906	1,248				0
*trnD*	13,907	13,963	57				124
*trnS2*	14,088	14,144	57				28
*trnQ*	14,173	14,231	59				11
*trnP*	14,243	14,303	61				5
*trnM*	14,309	14,371	63				0
*NCR3*	14,372	15,820	1,449				0
*rrnS*	15,821	16,727	907				0
*cox2*	16,728	17,429	702	233	ATA	TAA	2
*trnR*	17,432	17,487	56				58
*trnK*	17,546	17,608	63				42

**Notes.**

a Stop codons were not included.

nt nucleotide aa amino acid

The tRNA predicted secondary structures, ranging from 55 bp (*trnH*) to 63 bp (*trnI*, *trnK* and *trnM*) in size, are similar to those found in other nematode species (Fig. S1 and Table 2; See Kim et al., 2017 for details): 22 tRNAs lack a TΨC arm which is instead replaced by a TV-replacement loop, whereas *trnS1* and *trnS2* lack a dihydrouridine arm (DHU) and instead have a TΨC structure. Note that tRNAs encoding *trnE* and *trnK* are inferred to be duplicated: two nucleotide sequence regions, with respective sizes of 58 bp (*trnE*-1) and 62 bp (*trnE*-2), are inferred for *trnE* (Fig. S1 and Table 2). In addition, two *trnK* gene copies (consisting of 57 bp (*trnK*-1) and 63 bp (*trnK*-2)) are inferred. These duplicated tRNA copies are assumed to be functional, since they are predicted to form typical nematode tRNA secondary structures (Fig. S1). Duplication of tRNA genes has been occasionally reported from other nematode mitochondrial genomes such as *Camallanus cotti* (Zou et al., 2017), *Dracunculus medinensis* (Genbank accession no. NC_016019, Clark et al., 2016) and *Ruizia karukerae* (Kim et al., 2018).

The small ribosomal RNA (*rrnS*) is located between *NCR3* and *cox2*, with a length of 907 bp, and the large ribosomal RNA (*rrnL*) is located between *trnH* and *nad3*, with a length of 1,183 bp (Table 2). These two rRNA genes are among the largest in nematode mtDNA thus far reported. The predicted secondary structures of *rrnS* and *rrnL* of *A. varius* (Figs. S2 and S3) were inferred based on secondary structures of the rRNAs of other nematodes (*Caenorhabditids elegans* and *Ascaris suum* (Okimoto et al., 1992) and *Steinernema carpocapsae* (Montiel et al., 2006)), the 16S and 23S rRNAs of *Escherichia coli* (Dams et al., 1988; Gutell, 1994; Gutell, Gray & Schnare, 1993) and the LSU rRNA of *Tetrahymena pyriformis* (De Rijk et al., 1999). The secondary structures of the two rRNAs are generally conserved in nematode mitochondrial genomes, but some elements differ among nematode species. For example, helices 5 and 16 are absent from *rrnS* in *Dirofilaria immitis* and *Strongyloides stercoralis* (Hu, Chilton & Gasser, 2003; Hu et al., 2003), and helices C1, D6-10, D21, H3 and H4 are absent from *rrnL* in *Xiphinema americanum* (He et al., 2005). The predicted secondary structures of both rRNAs in the *A. varius* mt genome are similar to those reported from other nematodes such as *C. elegans* and *A. suum*. However, helices 35 and 39 and helices G9-G14 are predicted in *rrnS* and *rrnL*, respectively (Figs. S2 and S3). These elements are found in the 16S and 23S rRNAs of *E. coli*, but have not yet been reported from any other nematode species.

Three non-coding regions (NCRs) (ranging from 564 bp to 1,449 bp in size) are present in the *A. varius* mt genome, with high A+T content ranging from 80.5% (NCR1) to 83.7% (NCR3) (Fig. 1 and Table 2). Stem-and-loop structures formed by tandemly repeated sequences are found in all NCRs (Fig. S4).

### Mitochondrial genome phylogeny

**Figure 2:**
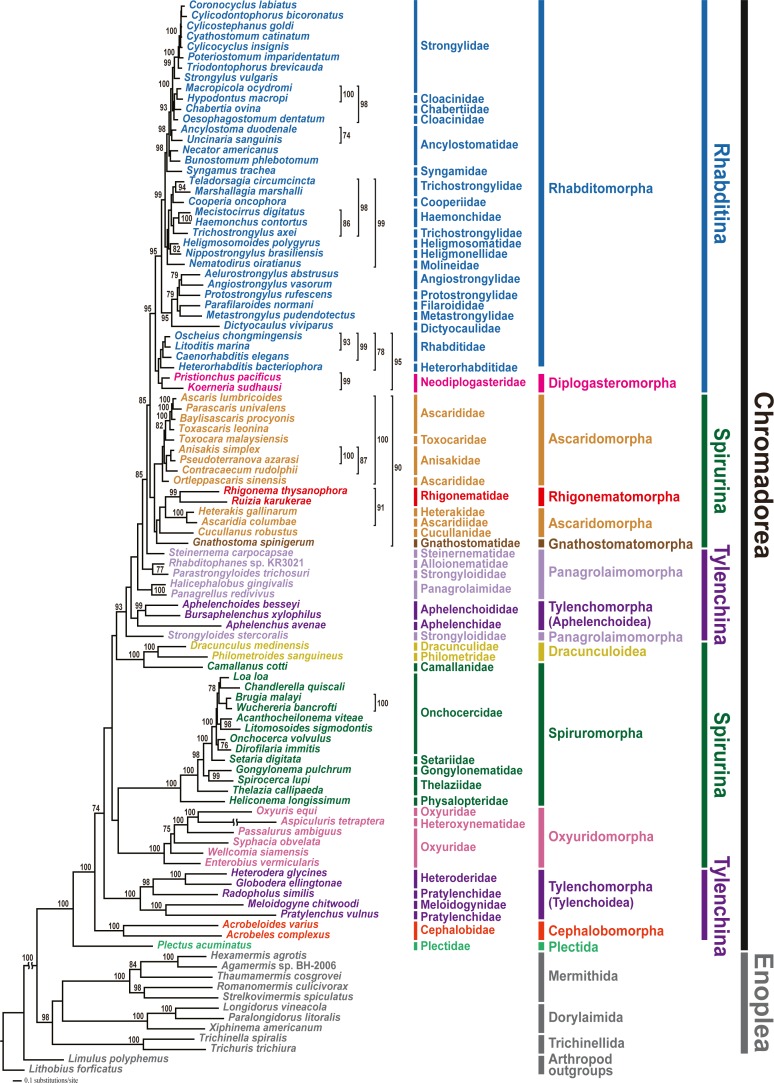
Maximum likelihood (ML) tree based on the amino acid sequences of 12 protein-coding genes from the mitochondrial genomes of 102 nematodes and two outgroups. Bootstrap percentages (BP) were calculated using the rapid bootstrapping method. BP values <70% are not shown.

Phylogenetic analyses involved two tree-building methods (ML and BI) for three different datasets (amino acid (AA) sequences, and nucleotide (NT) sequences with third codon positions either included or excluded) for all 12 PCGs from 102 nematode species. In all phylogenetic analyses, the respective monophyly of chromadorean and enoplean nematodes was strongly supported (Fig. 2 and Figs. S5–S9). Although supporting values among some chromadorean infraordinal groups were low, and some relationships varied depending on tree building methods and datasets, many elements of previously published mitogenome phylogenies were robustly supported: the non-monophyly of the suborder Spirurina, non-monophyly of Tylenchomorpha, and the nested position of Diplogasteromorpha within Rhabditomorpha (see Kim et al., 2017 for more details).

In this study, mitochondrial genome trees depicted the monophyly of two cephalobs (*A. varius* and *A. complexus*) with high branch support value (1.00 Bayesian posterior probability (BPP) in the BI tree and 100% bootstrap percentage (BP) in the ML tree) in all phylogenetic analyses (Fig. 2 and Figs. S5–S9). Cephalobomorpha represented by the two cephalob species (one partial and one complete sequence) was found to be basal to all chromadorean species (excluding *Plectus acuminatus* of Plectida) and separated from Panagrolaimomorpha and Tylenchomorpha, rendering Tylenchina non-monophyletic. In addition, we recently recognized that the *Panagrellus redivivus* mitochondrial genome sequence (GenBank accession #: KM192364) published in an earlier article (Kim et al., 2015) was incorrect, possibly due to PCR contamination with a different nematode species. In order to check its misidentification, we compared the mtDNA *cox1* and nuclear 28S rDNA sequences of the *P. redivivus* specimens used in Kim et al. (2015) with previously published and/or unpublished sequences of some Tylenchina species: the 28S rDNA sequence of the *P. redivivus* specimen of Kim et al. (2015) is very different (77.3% sequence identity) from the *P. redivivus* sequence (GenBank accession #: DQ145695; Nadler et al., 2006), but it is very similar to *A. complexus* (GenBank accession #: DQ145668; Nadler et al., 2006) (95.5% sequence identity) and *A. varius* sequence (99.5% sequence identity; GenBank accession #: MT233103 (this study)). The mtDNA *cox1* sequences between and *A. varius* (this study) and the *P. redivivus* specimens of Kim et al. (2015) differed by 9.6%. Based on this sequence comparison, the *P. redivivus* specimen reported in Kim et al. (2015) is assumed to be some Cephalobidae species, likely to be close to *Acrobeloides* species. Therefore, the mitochondrial genome sequence labelled as *P. redivivus* (*sensu* Kim et al., 2015) was retracted from Genbank and we replaced it with other *P. redivivus* sequence that is newly available on GenBank (GenBank accession no. AP017464.1; Clark et al., 2016). Unlike the non-sister relationship between *P. redivivus* and *Halicephalobus gingivalis* representing one of the Panagrolaimomorpha families (i.e., Panagrolaimidae) depicted from Kim et al. (2015), phylogenetic trees grouped the new *P. redivivus* sequence with *H. gingivalis* as a sister species (Fig. 2 and Figs. S5–S9). These relationships received high support values (1.00 BPP in BI tree and 93–100% BP in the ML tree). In previous phylogenetic studies, relationships among major groups within Tylenchina, including the position of Cephalobomorpha within Tylenchina, varied depending on the data source (i.e., morphology vs. molecular data) and taxon sampling used for phylogenetic inference. Traditional morphology-based hypotheses depicted a close relationship between cephalobomorphs and rhabditomorphs based on similarities in stoma and oesophagus features (Chitwood & Chitwood, 1977; Maggenti, 1981; Poinar, 1983). On the other hand, cephalobomorphs were inferred to be closely allied to tylenchs based on some morphological characters (Siddiqi, 1980; Siddiqi, 2000): a thickened internal cuticle with irregular basal bulb lumen of some tylenchomorphs was considered as a degenerate form of the valve in the basal bulb of cephalobomorphs; a common origin of the inverted U-shaped excretory system of cephalobomorphs and the asymmetrical system of tylenchomorphs; morphological similarity between phasmid-like structure in Tylenchidae species and hypodermal pores of some *Nothacrobeles* species; similarity of cuticle and lateral field in their structure; prodelphic reproductive system; reduction of papillae on the male tail. Moreover, in previous analyses of SSU rDNA, relationships among cephalobomorphs and other Tylenchina nematodes varied depending on dataset size (Blaxter et al., 1998; Holterman et al., 2006; Van Megen et al., 2009). The first phylum-wide nematode molecular phylogenies (Blaxter et al., 1998; Holterman et al., 2006) depicted Cephalobomorpha as sister to a Tylenchoidea + Aphelenchidae clade. In contrast, phylogenetic analysis with extended taxon coverage (van Megen et al., 2009) depicted Cephalobomorpha was sister to all Tylenchina species except Steinernematidae (Strongyloidoidea). Inconsistency in relationships among the groups within Tylenchina was interpreted as an artifact of long-branch attraction due to the high A+T contents of some aphelenchoids (Aphelenchoididae, Parasitaphelenchidae and Seinuridae) and the Panagrolaimomorpha (*ca* 54% and 57%, respectively; Holterman et al., 2006) (Holterman et al., 2006; Van Megen et al., 2009). Meanwhile, a molecular phylogenetic study of the suborder Cephalobina *sensu* (Andrássy, 1974) using LSU rDNA data (Nadler et al., 2006) depicted different relationships depending on analysis methods: a parsimonious tree showed a sister relationship between cephalobs and tylenchs, whereas an ML tree depicted a sister relationship between cephalobs and Panagrolaimidae (excluding *Brevibucca* sp.). Minimum evolution analysis found chambersiellids (infraorder Panagrolaimomorpha) to be the sister group of cephalobs. Such inconsistent relationships from different methods were assumed due to nucleotide compositional bias and/or long-branch attraction in LSU phylogeny (Nadler et al., 2006); thus, these relationships have needed validation using independent molecular markers and broader taxon sampling.

In the present study, mitochondrial genome trees from different methods strongly supported a paraphyletic relationship between Cephalobomorpha and Tylenchoidea of Tylenchomorpha, separate from Panagrolaimomorpha and Aphelenchoidea (another branch of Tylenchomorpha), rendering Tylenchina non-monophyletic. This result is not consistent with earlier studies including molecular phylogenies (SSU rDNA; Blaxter et al., 1998; Holterman et al., 2006) and morphology-based phylogenies (Siddiqi, 1980; Siddiqi, 2000) that supported close relationship between cephalobomorphs and tylenchs. The non-monophyly of Tylenchina inferred from mt genome trees contradicts previous SSU rDNA phylogenies that supported monophyly of Tylenchina members (Blaxter et al., 1998; Holterman et al., 2006; Van Megen et al., 2009). Although the present study strongly indicated non-monophyly of Tylenchina, relationships within Tylenchina are not fully resolved due to the instability of some non-monophyletic groups including Tylenchomorpha and Panagrolaimomorpha. Phylogenetic algorithms are not able to easily handle compositional heterogeneity across lineages, which may exist in both rDNA and mitochondrial genome markers (Liu et al., 2018; Yang et al., 2018). This factor limits the potential implications of phylogenetic estimation using mitochondrial genomes. However, it has been shown that mitochondrial genome composition across chromadorean species is not very heterogeneous (Kim et al., 2014). For better resolution of the phylogenetic relationships among Cephalobomorpha, Tylenchomorpha, and Panagrolaimomorpha, additional mitochondrial genome sequences from underrepresented taxa and/or further phylogenetic analyses using additional genetic markers are needed.

### Comparison of mitochondrial gene arrangement

Compared to enoplean species, the mitochondrial gene order of chromadorean nematodes is relatively conserved, and gene order similarity among certain groups has been interpreted as additional supporting evidence for their phylogenetic affinity, although an idiosyncratic pattern has also been reported in some species (Kim et al., 2014; Kim et al., 2017). Among nematode mt genomes reported thus far, gene arrangement patterns in most members of Rhabditomorpha (70 of 72 species), Ascaridomorpha (24 of 28 species), Diplogasteromorpha (one of two species), Aphelenchoididae (all three species except for *trnN*) and *Steinernema* (2 out of 4 species) are identical except for some idiosyncratic gene arrangements and a few minor translocations of tRNAs in some species (Jex et al., 2010; Kim et al., 2018; Liu et al., 2013; Montiel et al., 2006; Wang et al., 2016).

**Figure 3:**
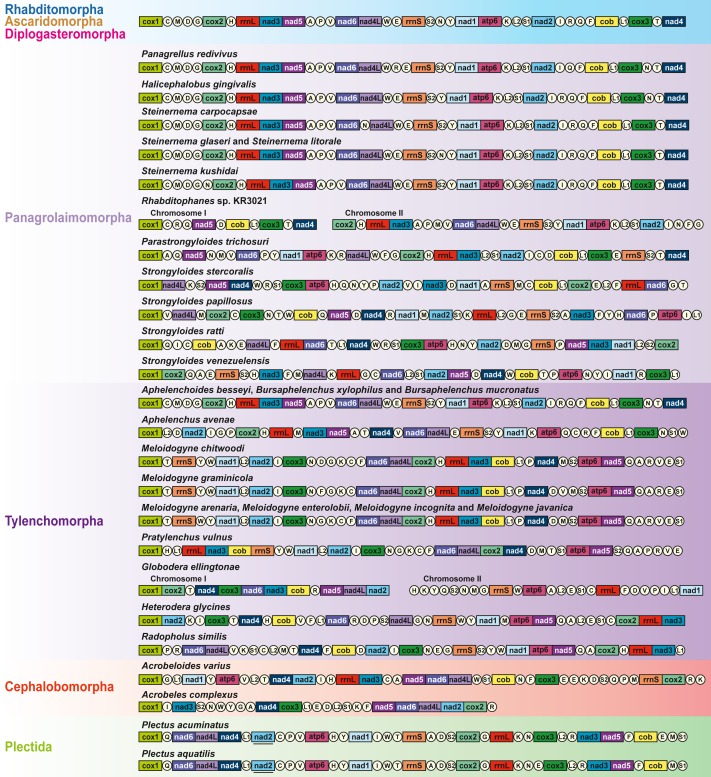
Linearized comparison of the gene arrangement of chromadorean nematode species including *Acrobeloides varius,* the first sequenced representative of Cephalobomorpha. All genes are encoded in the same direction and are not to scale. 22 tRNA genes are designated by a single letter amino acid code, based on the International Union of Pure and Applied Chemistry code. The two leucine and two serine tRNA genes are labeled according to their distinct anticodon sequences as L1 (*trnL1*-uag), L2 (*trnL2*-uaa), S1 (*trnS1*-ucu), and S2 (*trnS2*-uga). The non-coding regions are not included. Genes underlined in black (e.g., *nad2* in *Plectus* species) indicate the genes that are transcribed in a reverse direction. Bipartite mitochondrial genomes of *Rhabditophanes* sp. KR3021 and *Globodera ellingtonae* are presented as two split gene clusters, respectively.

A sister relationship between *A. varius* and *A. complexus* is recovered in phylogenetic analyses, but gene clusters shared between these two cephalobomorpha species (although the mtDNA of *A. complexus* is a partial sequence) are very limited: *nad5*+*nad6*+*nad4l* and *cox2*+*trnR*. The gene arrangement of *A. varius* is also very different from other Tylenchomorpha (Tylenchina) species, with very limited shared gene clusters (Fig. 3): *nad2*+*trnI*, *trnH*+*rrnL*+*nad3* and *nad6*+*nad4l* are shared with *Meloidogyne* spp. (Besnard et al., 2014; Humphreys-Pereira & Elling, 2014; Humphreys-Pereira & Elling, 2015), *nad2*+*trnI*, *rrnL+nad3*, *nad6+nad4l* and *trnS2*+*trnQ* with *Pratylenchus vulnus* (Sultana et al., 2013), *trnL1*+*nad1* and *trnT*+*nad4* with *Globodera ellingtonae* (Phillips et al., 2016), *trnT*+*nad4* and *rrnL*+*nad3* with *Heterodera glycines* (Gibson et al., 2011) and *nad6*+*nad4l*, *trnT*+*nad4*, *nad2*+*trnI* and *trnH*+*rrnL*+*nad3* with *R. similis* (Jacob et al., 2009). Shared gene clusters between *A. varius* and Panagrolaimomorpha species are also very limited: *trnT*+*nad4*, *trnH*+*rrnL*+*nad3*, *trnP*+*trnM*, *nad6*+*nad4L*+*trnW*, *nad2*+*trnI* and *trnN*+*trnF* are shared with *Rhabditophanes* sp. KR3021 (Hunt et al., 2016), *nad4L*+*trnW*, *trnH*+*rrnL*+*nad3*, *nad2*+*trnI*, *cox3*+*trnE* and *trnT*+*nad4* are shared with *Parastrongyloides trichosuri* (Hunt et al., 2016), *trnH*+*rrnL*+*nad3*, *nad4L*+*trnW*, *nad2*+*trnI* and *trnT*+*nad4* are shared with *S. carpocapsae* (Montiel et al., 2006) and *trnH*+*rrnL*+*nad3*, *nad6*+*nad4L*+*trnW*, *nad2*+*trnI* and *trnT*+*nad4* are shared with *S. glaseri*, *S. litorale*, *S. kushidai* (Kikuchi, Afrin & Yoshida, 2016), *H. gingivalis* (Kim et al., 2015) and *P. redivivus* (GenBank accession no. AP017464.1; Clark et al., 2016). Although gene arrangement of Tylenchina species is not conserved, the most common gene arrangement pattern of Rhabditomorpha, Ascaridomorpha and Diplogasteromorpha is also found in some Tylenchina species (Fig. 3): *Aphelenchoides besseyi*, *Bursaphelenchus xylophilus*, *B. muronatus* (Tylenchomorpha), *P. redivivus*, *H. gingivalis* and four *Steinernema* species (Panagrolaimomorpha), where gene order is almost identical and/or very similar to the most common gene arrangement type. Further mitochondrial genome survey of gene order information is needed to understand whether the gene order data can be useful to estimate phylogenetic relationships among Tylenchina species.

In conclusion, phylogenetic trees based on mitochondrial genome sequences indicate non-monophyly of Tylenchina (and a basal position of Cephalobomorpha to all chromadorean species excluding *Plectus*, separated from other Tylenchina members Panagrolaimomorpha and Tylenchomorpha). Nevertheless, phylogenetic relationships among Tylenchina nematodes are not clearly established due to inconsistent placement of some taxa including panagrolaimomorphs and aphelenchids. Along with employing independent molecular markers, further mitochondrial genome information from the underrepresented taxa of Panagrolaimomorpha and Cephalobomorpha needs to be obtained to clarify unresolved relationships within Tylenchina.

##  Supplemental Information

10.7717/peerj.9108/supp-1 Table S1 Species list, classification, and GenBank accession numbers of 102 nematode species and two arthropod species used for phylogenetic analyses in this study

10.7717/peerj.9108/supp-2 Table S2 The best-fit models estimated from each of the 12 PCGs of 102 nematodes and two arthropods

10.7717/peerj.9108/supp-3 Table S3 Nucleotide composition of the mitochondrial genome of *Acrobeloides varius*

10.7717/peerj.9108/supp-4 Table S4 Genetic code and codon usage for the mitochondrial protein coding genes of *Acrobeloides varius*

10.7717/peerj.9108/supp-5 Figure S1 Putative secondary structures of 24 tRNAs of *Acrobeloides varius* mitochondrial genome

10.7717/peerj.9108/supp-6 Figure S2 Putative secondary structures of *rrnS* in *Acrobeloides varius* mitochondrial genome Watson-Crick base pairings are denoted by lines and G-U pairs are indicated by dots. The numbers identify the conserved secondary structure elements defined by Dams et al. (1988).

10.7717/peerj.9108/supp-7 Figure S3 Putative secondary structures of *rrnL* in *Acrobeloides varius* mitochondrial genome Watson-Crick base pairings are denoted by lines and G-U pairs are indicated by dots. The numbers identify the conserved secondary structure elements defined by De Rijk et al. (1999).

10.7717/peerj.9108/supp-8 Figure S4 Hypothesized stem-and-loop secondary structures in non-coding regions of *Acrobeloides varius* mitochondrial genome

10.7717/peerj.9108/supp-9 Figure S5 Maximum likelihood tree based on the nucleotide sequences of 12 protein-coding genes from the mitochondrial genomes of 102 nematodes and 2 outgroups Bootstrap percentages (BP) were calculated using the rapid bootstrapping method. BP values <70% are not shown.

10.7717/peerj.9108/supp-10 Figure S6 Maximum likelihood tree based on the nucleotide sequences (excluding 3rd codon positions) of 12 protein-coding genes from the mitochondrial genomes of 102 nematodes and 2 outgroups Bootstrap percentages (BP) were calculated using the rapid bootstrapping method. BP values <70% are not shown.

10.7717/peerj.9108/supp-11 Figure S7 Bayesian inference tree based on the amino acid sequences of 12 protein-coding genes from the mitochondrial genomes of 102 nematodes and 2 outgroups Bayesian posterior probabilities (BPP) were estimated after discarding the initial 250 trees (the first 25 × 10^4^ generations) as burn-in. BPP values <0.7 are not shown.

10.7717/peerj.9108/supp-12 Figure S8 Bayesian inference tree based on the nucleotide sequences of 12 protein-coding genes from the mitochondrial genomes of 102 nematodes and 2 outgroups Bayesian posterior probabilities (BPP) were estimated after discarding the initial 250 trees (the first 25 × 10^4^ generations) as burn-in. BPP values <0.7 are not shown.

10.7717/peerj.9108/supp-13 Figure S9 Bayesian inference tree based on the nucleotide sequences (excluding 3rd codon positions) of 12 protein-coding genes from the mitochondrial genomes of 102 nematodes and 2 outgroups Bayesian posterior probabilities (BPP) were estimated after discarding the initial 250 trees (the first 25 × 10^4^ generations) as burn-in. BPP values <0.7 are not shown.

## Additional Information and Declarations

### Data Availability

The following information was supplied regarding data availability: The mt genome sequence and the 28S sequence of *Acrobeloides varius* are available at GenBank: MK559448 and MT233103.
