# Supplementary material for: The mitochondrial genome of Acrobeloides varius (Cephalobomorpha) confirms non-monophyly of Tylenchina (Nematoda)
Source: PeerJ. 2020 May 13;8:e9108. doi: 10.7717/peerj.9108 (PMC7229770; doi:10.7717/peerj.9108)
Supplement: Table S1 [file peerj-08-9108-s001.docx]

**Supplemental Table S1:**

**Species list, classification, and GenBank accession numbers of 102 nematode species and two arthropod species used for phylogenetic analyses in this study**

| Species | Classification^*^ | GenBank accession no. |
| --- | --- | --- |
| *Acanthocheilonema viteae* | Chromadorea; Spiruromorpha; Onchocercidae | NC_016197 |
| *Acrobeles complexus* | Chromadorea; Cephalobomorpha; Cephalobidae | KM192361.1 |
| *Acrobeloides varius* | Chromadorea; Cephalobomorpha; Cephalobidae | This study |
| *Aelurostrongylus abstrusus* | Chromadorea; Rhabditomorpha; Angiostrongylidae | NC_019571 |
| *Agamermis* sp. BH-2006 | Enoplea; Mermithida; Mermithidae | NC_008231 |
| *Ancylostoma duodenale* | Chromadorea; Rhabditomorpha; Ancylostomatidae | NC_003415 |
| *Angiostrongylus vasorum* | Chromadorea; Rhabditomorpha; Angiostrongylidae | NC_018602 |
| *Anisakis simplex* | Chromadorea; Ascaridomorpha; Anisakidae | NC_007934 |
| *Aphelenchoides besseyi* | Chromadorea; Tylenchomorpha; Aphelenchoididae | NC_025291 |
| *Aphelenchus avenae* | Chromadorea; Tylenchomorpha; Aphelenchidae | KM192362.1 |
| *Ascaridia columbae* | Chromadorea; Ascaridomorpha; Ascaridiidae | NC_021643 |
| *Ascaris lumbricoides* | Chromadorea; Ascaridomorpha; Ascarididae | NC_016198 |
| *Aspiculuris tetraptera* | Chromadorea; Oxyuridomorpha;Heteroxynematidae | KT764937 |
| *Baylisascaris procyonis* | Chromadorea; Ascaridomorpha; Ascarididae | NC_016200 |
| *Brugia malayi* | Chromadorea; Spiruromorpha; Onchocercidae | NC_004298 |
| *Bunostomum phlebotomum* | Chromadorea; Rhabditomorpha; Ancylostomatidae | NC_012308 |
| *Bursaphelenchus xylophilus* | Chromadorea; Tylenchomorpha; Aphelenchoididae | NC_023208 |
| *Caenorhabditis elegans* | Chromadorea; Rhabditomorpha; Rhabditidae | NC_001328 |
| *Camallanus cotti* | Chromadorea; Spiruromorpha; Camallanidae | NC_036308.1 |
| *Chabertia ovina* | Chromadorea; Rhabditomorpha; Chabertiidae | NC_013831 |
| *Chandlerella quiscali* | Chromadorea; Spiruromorpha; Onchocercidae | NC_014486 |
| *Contracaecum rudolphii* | Chromadorea; Ascaridomorpha; Anisakidae | NC_014870 |
| *Cooperia oncophora* | Chromadorea; Rhabditomorpha; Cooperiidae | NC_004806 |
| *Coronocyclus labiatus* | Chromadorea; Rhabditomorpha; Strongylidae | NC_042234.1 |
| *Cucullanus robustus* | Chromadorea; Ascaridomorpha; Cucullanidae; | NC_016128 |
| *Cyathostomum catinatum* | Chromadorea; Rhabditomorpha; Strongylidae | NC_035003.1 |
| *Cylicocyclus insigne* | Chromadorea; Rhabditomorpha; Strongylidae | NC_013808 |
| *Cylicodontophorus bicoronatus* | Chromadorea; Rhabditomorpha; Strongylidae | NC_042141.1 |
| *Cylicostephanus goldi* | Chromadorea; Rhabditomorpha; Strongylidae | AP017681 |
| *Dictyocaulus viviparus* | Chromadorea; Rhabditomorpha; Dictyocaulidae | NC_019810 |
| *Dirofilaria immitis* | Chromadorea; Spiruromorpha; Onchocercidae | NC_005305 |
| *Dracunculus medinensis* | Chromadorea; Dracunculoidea; Dracunculidae | NC_016019 |
| *Enterobius vermicularis* | Chromadorea; Oxyuridomorpha; Oxyuridae | NC_011300 |
| *Globodera ellingtonae* | Chromadorea; Tylenchomorpha; Heteroderidae | KU726971, KU726972 |
| *Gnathostoma spinigerum* | Chromadorea; Gnathostomatomorpha; Gnathostomatidae | NC_027726 |
| *Gongylonema pulchrum* | Chromadorea; Spiruromorpha; Gongylonematidae | NC_026687 |
| *Haemonchus contortus* | Chromadorea; Rhabditomorpha; Haemonchidae | NC_010383 |
| *Halicephalobus gingivalis* | Chromadorea; Panagrolaimomorpha; Panagrolaimidae | KM192363.1 |
| *Heliconema longissimum* | Chromadorea; Spiruromorpha; Physalopteridae | NC_016127 |
| *Heligmosomoides polygyrus* | Chromadorea; Rhabditomorpha; Heligmosomatidae | AP017688 |
| *Heterakis gallinarum* | Chromadorea; Ascaridomorpha; Heterakidae | NC_029839 |
| *Heterodera glycines* | Chromadorea; Tylenchomorpha; Heteroderidae | HM640930.1 |
| *Heterorhabditis bacteriophora* | Chromadorea; Rhabditomorpha; Heterorhabditidae | NC_008534 |
| *Hexamermis agrotis* | Enoplea; Mermithida; Mermithidae | NC_008828 |
| *Hypodontus macropi* | Chromadorea; Rhabditomorpha; Cloacinidae | NC_023098 |
| *Koerneria sudhausi* | Chromadorea; Diplogasteromorpha; Neodiplogasteridae | NC_029233 |
| *Litoditis* aff. *marina* | Chromadorea; Rhabditomorpha; Rhabditidae | NC_027694.1 |
| *Litomosoides sigmodontis* | Chromadorea; Spiruromorpha; Onchocercidae | AP017689 |
| *Loa loa* | Chromadorea; Spiruromorpha; Onchocercidae | NC_016199 |
| *Longidorus vineacola* | Enoplea; Dorylaimida; Longidoridae | NC_033867.1 |
| *Macropicola ocydromi* | Chromadorea; Rhabditomorpha; Strongylidae | NC_023099 |
| *Marshallagia marshalli* | Chromadorea; Rhabditomorpha; Trichostrongylidae | NC_036409.1 |
| *Mecistocirrus digitatus* | Chromadorea; Rhabditomorpha; Haemonchidae | NC_013848 |
| *Meloidogyne chitwoodi* | Chromadorea; Tylenchomorpha; Meloidogynidae | NC_024096 |
| *Metastrongylus pudendotectus* | Chromadorea; Rhabditomorpha; Metastrongylidae | NC_013813 |
| *Necator americanus* | Chromadorea; Rhabditomorpha; Ancylostomatidae | NC_003416 |
| *Nematodirus oiratianus* | Chromadorea; Rhabditomorpha; Molineidae | NC_024639 |
| *Nippostrongylus brasiliensis* | Chromadorea; Rhabditomorpha; Heligmonellidae | NC_033886.1 |
| *Oesophagostomum dentatum* | Chromadorea; Rhabditomorpha; Cloacinidae | NC_013817 |
| *Onchocerca volvulus* | Chromadorea; Spiruromorpha; Onchocercidae | NC_001861 |
| *Ortleppascaris sinensis* | Chromadorea; Ascaridomorpha; Ascarididae | NC_036669.1 |
| *Oscheius chongmingensis* | Chromadorea; Rhabditomorpha; Rhabditidae | KP257594 |
| *Oxyuris equi* | Chromadorea; Oxyuridomorpha; Oxyuridae | NC_027190 |
| *Panagrellus redivivus* | Chromadorea; Panagrolaimomorpha; Panagrolaimidae | AP017464.1 |
| *Parafilaroides normani* | Chromadorea; Rhabditomorpha; Filaroididae | NC_024656 |
| *Paralongidorus litoralis* | Enoplea; Dorylaimida; Longidoridae | NC_033868.1 |
| *Parascaris univalens* | Chromadorea; Ascaridomorpha; Ascarididae | NC_024884 |
| *Parastrongyloides trichosuri* | Chromadorea; Panagrolaimomorpha; Strongyloididae | LC050209 |
| *Passalurus ambiguus* | Chromadorea; Oxyuridomorpha; Oxyuridae | NC_028345 |
| *Philometroides sanguineus* | Chromadorea; Dracunculoidea; Philometridae | NC_024931 |
| *Plectus acuminatus* | Chromadorea; Plectida; Plectidae | KX017523 |
| *Poteriostomum imparidentatum* | Chromadorea; Rhabditomorpha; Strongylidae | NC_035005.1 |
| *Pratylenchus vulnus* | Chromadorea; Tylenchomorpha; Pratylenchidae | NC_020434 |
| *Pristionchus pacificus* | Chromadorea; Diplogasteromorpha; Neodiplogasteridae | NC_015245 |
| *Protostrongylus rufescens* | Chromadorea; Rhabditomorpha; Protostrongylidae | NC_023262 |
| *Pseudoterranova azarasi* | Chromadorea; Ascaridomorpha; Anisakidae | NC_027163 |
| *Radopholus similis* | Chromadorea; Tylenchomorpha; Pratylenchidae | NC_013253 |
| *Rhabditophanes* sp. KR3021 | Chromadorea; Panagrolaimomorpha; Alloionematidae | LC050214.1, C050215.1 |
| *Rhigonema thysanophora* | Chromadorea; Rhigonematomorpha; Rhigonematidae | NC_024020 |
| *Romanomermis culicivorax* | Enoplea; Mermithida; Mermithidae | NC_008640 |
| *Ruizia karukerae* | Chromadorea; Rhigonematomorpha; Rhigonematidae | MF509850, MF509851 |
| *Setaria digitata* | Chromadorea; Spiruromorpha; Setariidae | NC_014282 |
| *Spirocerca lupi* | Chromadorea; Spiruromorpha; Thelaziidae | NC_021135 |
| *Steinernema carpocapsae* | Chromadorea; Panagrolaimomorpha; Steinernematidae | NC_005941 |
| *Strelkovimermis spiculatus* | Enoplea; Mermithida; Mermithidae | NC_008047 |
| *Strongyloides stercoralis* | Chromadorea; Panagrolaimomorpha; Strongyloididae | NC_028624 |
| *Strongylus vulgaris* | Chromadorea; Rhabditomorpha; Strongylidae | NC_013818 |
| *Syngamus trachea* | Chromadorea; Rhabditomorpha; Syngamidae | NC_013821 |
| *Syphacia obvelata* | Chromadorea; Oxyuridomorpha; Oxyuridae | NC_029239 |
| *Teladorsagia circumcincta* | Chromadorea; Rhabditomorpha; Trichostrongylidae | NC_013827 |
| *Thaumamermis cosgrovei* | Enoplea; Mermithida; Mermithidae | NC_008046 |
| *Thelazia callipaeda* | Chromadorea; Spiruromorpha; Thelaziidae | NC_018363 |
| *Toxascaris leonina* | Chromadorea; Ascaridomorpha; Ascarididae | NC_023504 |
| *Toxocara malaysiensis* | Chromadorea; Ascaridomorpha; Toxocaridae | NC_010527 |
| *Trichinella spiralis* | Enoplea; Trichocephalida; Trichinellidae | NC_002681 |
| *Trichostrongylus axei* | Chromadorea; Rhabditomorpha; Trichostrongylidae | NC_013824 |
| *Trichuris trichiura* | Enoplea; Trichocephalida; Trichuridae | NC_017750 |
| *Triodontophorus brevicauda* | Chromadorea; Rhabditomorpha; Strongylidae | NC_026729 |
| *Uncinaria sanguinis* | Chromadorea; Rhabditomorpha; Ancylostomatidae | NC_025267 |
| *Wellcomia siamensis* | Chromadorea; Oxyuridomorpha; Oxyuridae | NC_016129 |
| *Wuchereria bancrofti* | Chromadorea; Spiruromorpha; Onchocercidae | NC_016186 |
| *Xiphinema americanum* | Enoplea; Dorylaimida; Longidoridae | NC_005928 |
| *Limulus polyphemus* | Arthropoda (outgroup) | NC_003057 |
| *Lithobius forficatus* | Arthropoda (outgroup) | NC_002629 |

^*^Classification modified from De Ley and Blaxter (2002)
