# Supplementary material for: The mitochondrial genome of Acrobeloides varius (Cephalobomorpha) confirms non-monophyly of Tylenchina (Nematoda)
Source: PeerJ. 2020 May 13;8:e9108. doi: 10.7717/peerj.9108 (PMC7229770; doi:10.7717/peerj.9108)
Supplement: Table S2 [file peerj-08-9108-s002.docx]

**Supplemental Table S2:**

**The best-fit models estimated from each of the 12 PCGs of 102 nematodes and two arthropods**

| **gene** | **MrModeltest for nucleotide** | **MrModeltest for nucleotide without thrid codon position** | **ProtTest for amino acid** |
| --- | --- | --- | --- |
| *atp6* | GTR+I+G | GTR+I+G | JTT+G+F |
| *cob* | GTR+I+G | GTR+I+G | MtArt+I+G+F |
| *cox1* | GTR+I+G | GTR+I+G | MtArt+I+G+F |
| *cox2* | GTR+I+G | GTR+I+ G | LG+G+F |
| *cox3* | GTR+I+G | GTR+ G | MtArt+I+G+F |
| *nad1* | GTR+I+G | GTR+I+G | MtArt+I+G+F |
| *nad2* | GTR+I+G | GTR+I+G | MtArt+I+G+F |
| *nad3* | GTR+I+G | GTR+G | JTT+G+F |
| *nad4* | GTR+G | GTR+I+G | MtArt+I+G+F |
| *nad4l* | GTR+G | GTR+G | JTT+G+F |
| *nad5* | GTR+G | GTR+G | MtArt+I+G+F |
| *nad6* | GTR+I+G | GTR+G | Vt+I+G+F |
