# Supplementary material for: The mitochondrial genome of Acrobeloides varius (Cephalobomorpha) confirms non-monophyly of Tylenchina (Nematoda)
Source: PeerJ. 2020 May 13;8:e9108. doi: 10.7717/peerj.9108 (PMC7229770; doi:10.7717/peerj.9108)
Supplement: Table S3 [file peerj-08-9108-s003.docx]

**Supplemental Table S3:**

**Nucleotide composition of the mitochondrial genome of *Acrobeloides varius***

| Nucleotide | Length (bp) | T (%) | C (%) | A (%) | G (%) | A+T (%) | G+C (%) |
| --- | --- | --- | --- | --- | --- | --- | --- |
| Entire sequence | 17,650 | 45.7 | 8.8 | 30.9 | 14.6 | 76.6 | 23.4 |
| Protein coding sequence | 10,170 | 47.6 | 8.8 | 28.8 | 14.8 | 76.4 | 23.6 |
| Codon position* |  |  |  |  |  |  |  |
| 1st | 3,390 | 39.7 | 9.2 | 32.1 | 19.0 | 71.8 | 28.2 |
| 2nd | 3,390 | 50.7 | 13.6 | 19.4 | 16.3 | 70.1 | 29.9 |
| 3rd | 3,390 | 52.4 | 3.6 | 34.9 | 9.1 | 87.3 | 12.7 |
| Ribosomal RNA gene sequence | 2,090 | 38.0 | 11.9 | 32.6 | 17.5 | 70.6 | 29.4 |
| Transfer RNA gene sequence | 1,404 | 36.8 | 12.3 | 31.6 | 19.2 | 68.4 | 31.6 |
| Non-coding region | 3,261 | 47.4 | 6.5 | 34.7 | 11.4 | 82.1 | 17.9 |

*Termination codons were not included
