# Supplementary material for: The mitochondrial genome of Acrobeloides varius (Cephalobomorpha) confirms non-monophyly of Tylenchina (Nematoda)
Source: PeerJ. 2020 May 13;8:e9108. doi: 10.7717/peerj.9108 (PMC7229770; doi:10.7717/peerj.9108)
Supplement: Table S4 [file peerj-08-9108-s004.docx]

**Supplemental Table S4:**

**Genetic code and codon usage for the mitochondrial protein coding genes of *Acrobeloides varius***

| **Codon** | **AA** | **No.** | **%** | **Codon** | **AA** | **No.** | **%** |
| --- | --- | --- | --- | --- | --- | --- | --- |
| TTT | Phe | 359 | 10.6 | TAT | Tyr | 184 | 5.4 |
| TTC | Phe | 34 | 1.0 | TAC | Tyr | 13 | 0.4 |
| TTA | Leu | 380 | 11.2 | TAA | * | 8 | 0.2 |
| TTG | Leu | 87 | 2.6 | TAG | * | 4 | 0.1 |
| CTT | Leu | 48 | 1.4 | CAT | His | 62 | 1.8 |
| CTC | Leu | 11 | 0.3 | CAC | His | 4 | 0.1 |
| CTA | Leu | 25 | 0.7 | CAA | Gln | 36 | 1.1 |
| CTG | Leu | 6 | 0.2 | CAG | Gln | 10 | 0.3 |
| ATT | Ile | 254 | 7.5 | AAT | Asn | 134 | 3.9 |
| ATC | Ile | 10 | 0.3 | AAC | Asn | 10 | 0.3 |
| ATA | Met | 182 | 5.3 | AAA | Lys | 55 | 1.6 |
| ATG | Met | 63 | 1.9 | AAG | Lys | 38 | 1.1 |
| GTT | Val | 146 | 4.3 | GAT | Asp | 24 | 0.7 |
| GTC | Val | 9 | 0.3 | GAC | Asp | 1 | 0.0 |
| GTA | Val | 83 | 2.4 | GAA | Glu | 61 | 1.8 |
| GTG | Val | 22 | 0.6 | GAG | Glu | 25 | 0.7 |
| TCT | Ser | 125 | 3.7 | TGT | Cys | 26 | 0.8 |
| TCC | Ser | 1 | 0.0 | TGC | Cys | 3 | 0.1 |
| TCA | Ser | 45 | 1.3 | TGA | Trp | 75 | 2.2 |
| TCG | Ser | 8 | 0.2 | TGG | Trp | 5 | 0.1 |
| CCT | Pro | 51 | 1.5 | CGT | Arg | 20 | 0.6 |
| CCC | Pro | 4 | 0.1 | CGC | Arg | 3 | 0.1 |
| CCA | Pro | 21 | 0.6 | CGA | Arg | 11 | 0.3 |
| CCG | Pro | 0 | 0.0 | CGG | Arg | 1 | 0.0 |
| ACT | Thr | 91 | 2.7 | AGT | Ser | 98 | 2.9 |
| ACC | Thr | 2 | 0.1 | AGC | Ser | 8 | 0.2 |
| ACA | Thr | 28 | 0.8 | AGA | Ser | 93 | 2.7 |
| ACG | Thr | 5 | 0.1 | AGG | Ser | 16 | 0.5 |
| GCT | Ala | 54 | 1.6 | GGT | Gly | 100 | 2.9 |
| GCC | Ala | 3 | 0.1 | GGC | Gly | 7 | 0.2 |
| GCA | Ala | 21 | 0.6 | GGA | Gly | 68 | 2.0 |
| GCG | Ala | 2 | 0.1 | GGG | Gly | 19 | 0.6 |

*Termination codon
