# Supplementary figures and images for: The mitochondrial genome of Acrobeloides varius (Cephalobomorpha) confirms non-monophyly of Tylenchina (Nematoda)
Source: PeerJ. 2020 May 13;8:e9108. doi: 10.7717/peerj.9108 (PMC7229770; doi:10.7717/peerj.9108)

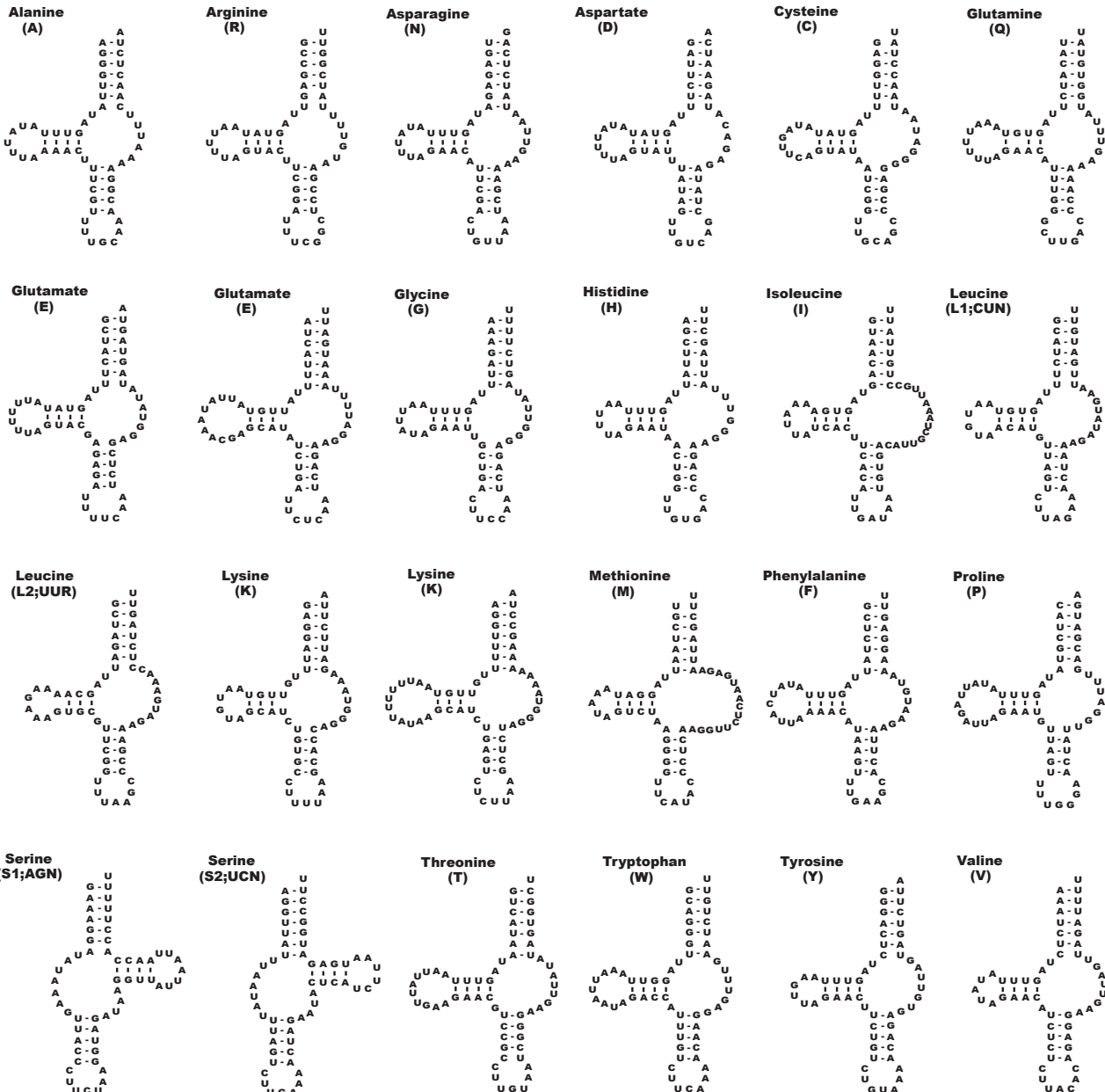

Supplement: Figure S1 [file peerj-08-9108-s005.pdf]

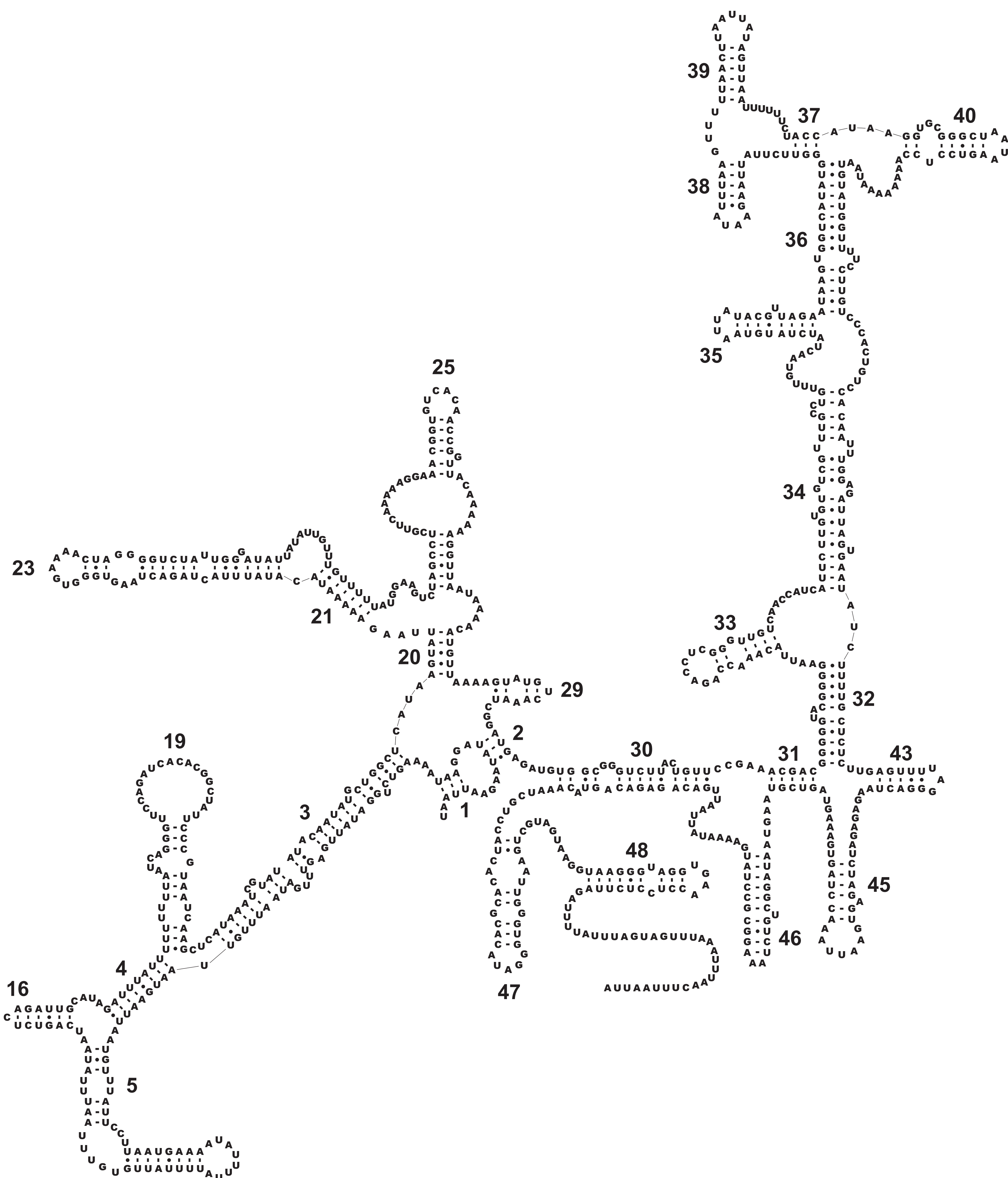

Supplement: Figure S2 — Watson-Crick base pairings are denoted by lines and G-U pairs are indicated by dots. The numbers identify the conserved secondary structure elements defined by Dams et al. (1988). [file peerj-08-9108-s006.pdf]

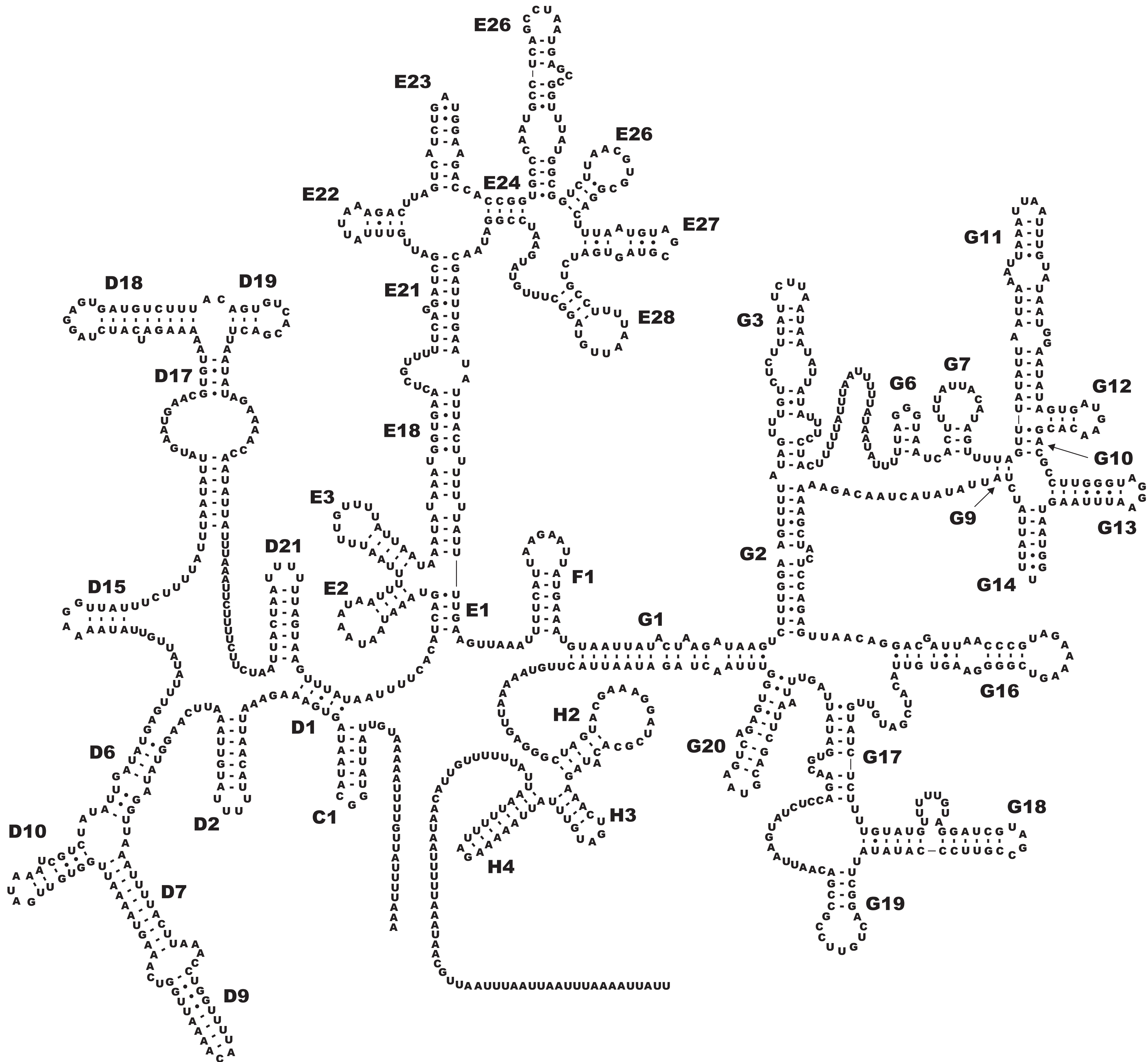

Supplement: Figure S3 — Watson-Crick base pairings are denoted by lines and G-U pairs are indicated by dots. The numbers identify the conserved secondary structure elements defined by De Rijk et al. (1999). [file peerj-08-9108-s007.pdf]
