# Supplementary material for: The mitochondrial genome of Acrobeloides varius (Cephalobomorpha) confirms non-monophyly of Tylenchina (Nematoda)
Source: PeerJ. 2020 May 13;8:e9108. doi: 10.7717/peerj.9108 (PMC7229770; doi:10.7717/peerj.9108)
Supplement: Figure S4 [file peerj-08-9108-s008.pdf]

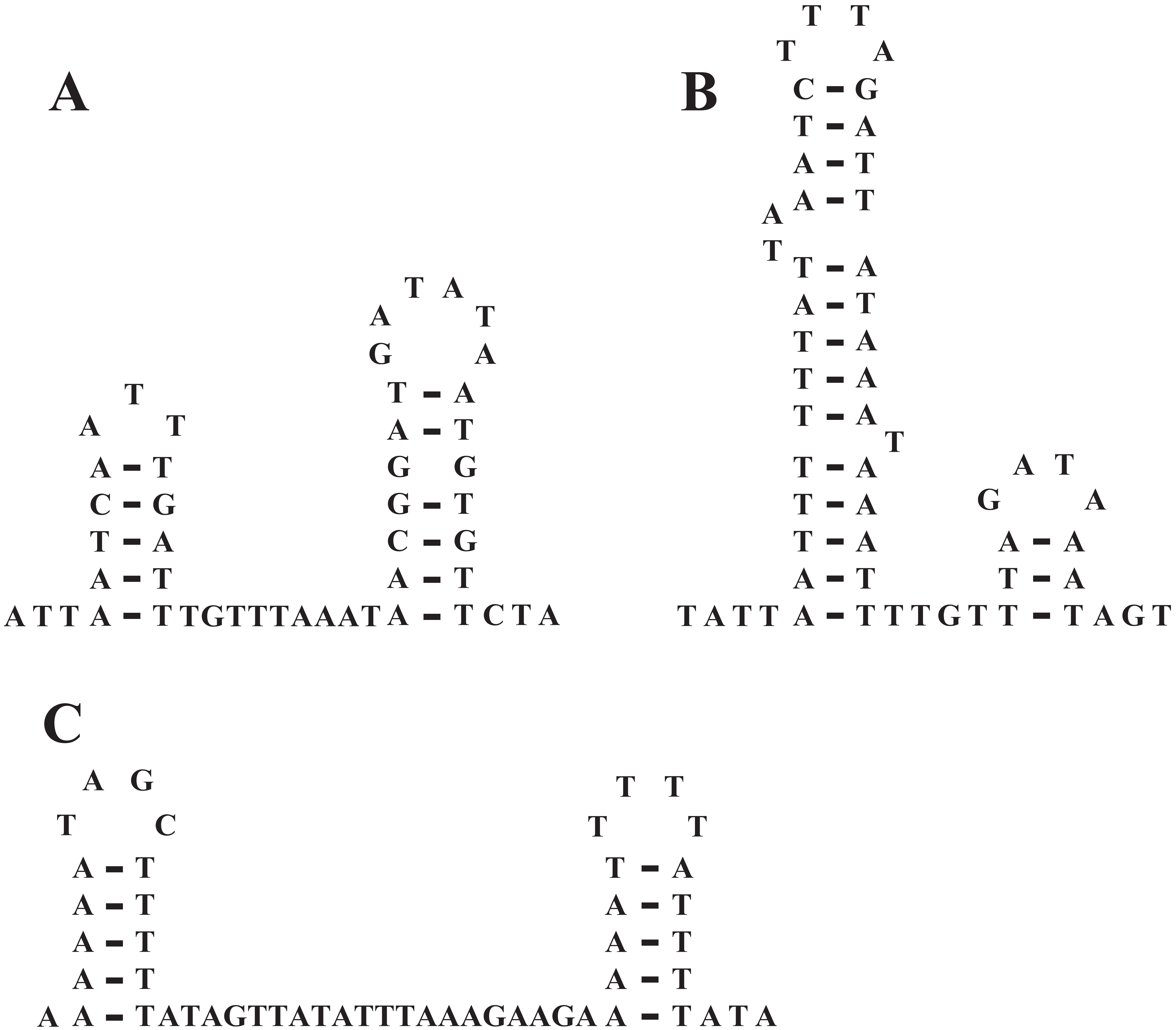
A
B
C
ATTA-TTGTTTAAATA-TCTA
TATTA-TTTGTT-TAGT
AA-TATAGTTATATTTAAAGAAGAA-TATA
