# Supplementary material for: The mitochondrial genome of Acrobeloides varius (Cephalobomorpha) confirms non-monophyly of Tylenchina (Nematoda)
Source: PeerJ. 2020 May 13;8:e9108. doi: 10.7717/peerj.9108 (PMC7229770; doi:10.7717/peerj.9108)
Supplement: Figure S5 — Bootstrap percentages (BP) were calculated using the rapid bootstrapping method. BP values <70% are not shown. [file peerj-08-9108-s009.pdf]

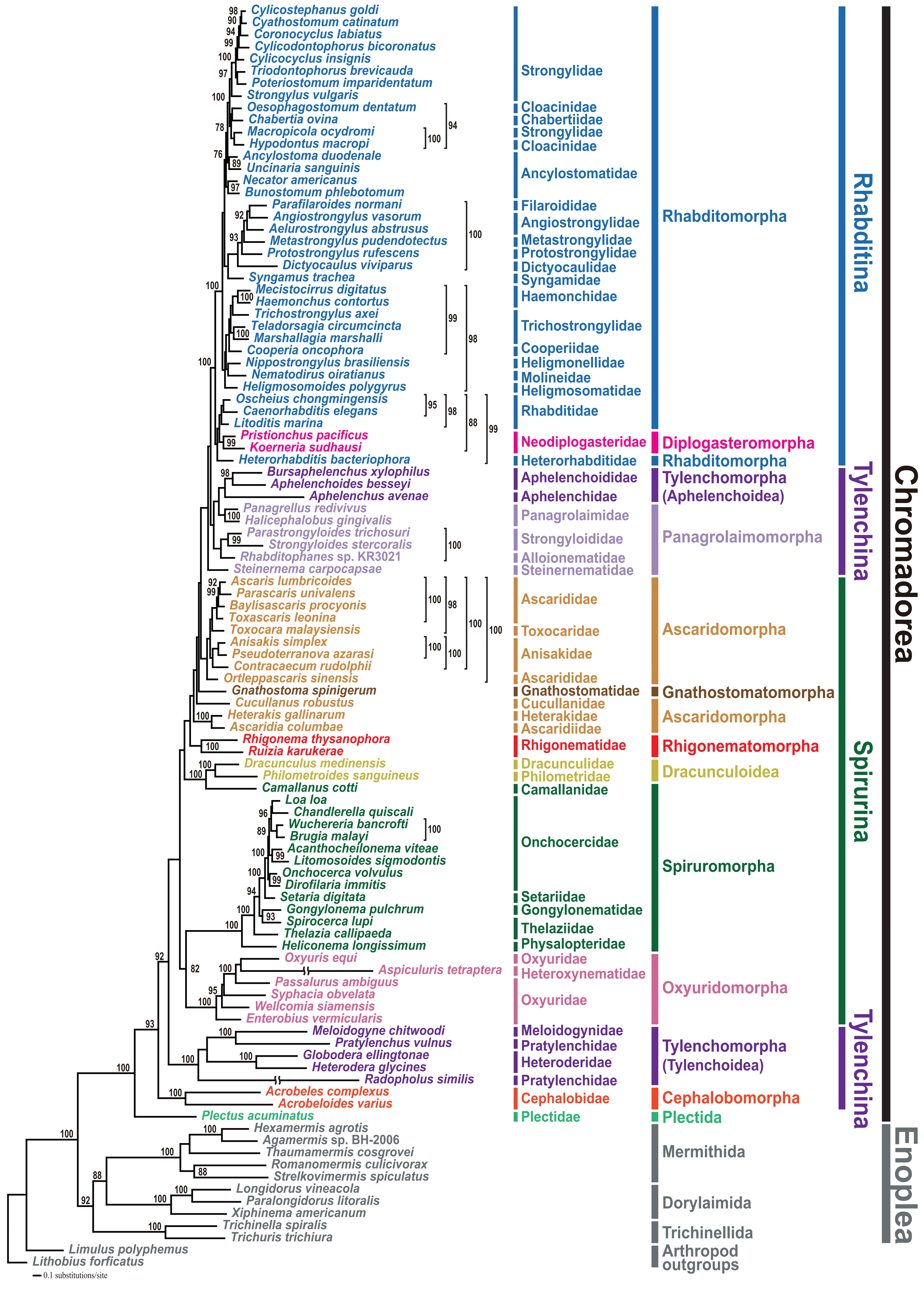

Cylicostephanus goldi
Cyathostomum catinatum
Coronocyclus labiatus
Cylicodontophorus bicoronatus
Cylicocyclus insignis
Triodontophorus brevicauda
Poteriostomum imparidentatum
Strongylus vulgaris
Oesophagostomum dentatum
Chabertia ovina
Macropicola ocydromi
Hypodontus macropi
Ancylostoma duodenale
Uncinaria sanguinis
Necator americanus
Bunostomum phlebotomum
Parafilaroides normani
Angiostrongylus vasorum
Aelurostrongylus abstrusus
Metastrongylus pudendotectus
Protostrongylus rufescens
Dictyocaulus viviparus
Syngamus trachea
Mecistocirrus digitatus
Haemonchus contortus
Trichostrongylus axei
Teladorsagia circumcincta
Marshallagia marshalli
Cooperia oncophora
Nippostrongylus brasiliensis
Nematodirus oiratianus
Heligmosomoides polygyrus
Oscheius chongmingensis
Caenorhabditis elegans
Litoditis marina
Pristionchus pacificus
Koerneria sudhausi
Heterorhabditis bacteriophora
Bursaphelenchus xylophilus
Aphelenchoides besseyi
Aphelenchus avenae
Panagrellus redivivus
Halicephalobus gingivalis
Parastrongyloides trichosuri
Strongyloides stercoralis
Rhabditophanes sp. KR3021
Steinernema carpocapsae
Ascaris lumbricoides
Parascaris univalens
Baylisascaris procyonis
Toxascaris leonina
Toxocara malaysiensis
Anisakis simplex
Pseudoterranova azarasi
Contracaecum rudolphii
Ortleppascaris sinensis
Gnathostoma spinigerum
Cucullanus robustus
Heterakis gallinarum
Ascaridia columbae
Rhigonema thysanophora
Ruizia karukerae
Dracunculus medinensis
Philometroides sanguineus
Camallanus cotti
Loa loa
Chandlerella quiscali
Wuchereria bancrofti
Brugia malayi
Acanthocheilonema viteae
Litomosoides sigmodontis
Onchocerca volvulus
Dirofilaria immitis
Setaria digitata
Gongylonema pulchrum
Spirocerca lupi
Thelazia callipaeda
Heliconema longissimum
Oxyuris equi
Aspiculuris tetraptera
Passalurus ambiguus
Syphacia obvelata
Wellcomia siamensis
Enterobius vermicularis
Meloidogyne chitwoodi
Pratylenchus vulnus
Globodera ellingtonae
Heterodera glycines
Radopholus similis
Acrobeles complexus
Acrobeloides varius
Plectus acuminatus
Hexamermis agrotis
Agamermis sp. BH-2006
Thaumamermis cosgrovei
Romanomermis culicivorax
Strelkovimermis spiculatus
Longidorus vineacola
Paralongidorus litoralis
Xiphinema americanum
Trichinella spiralis
Trichuris trichiura
Limulus polyphemus
Lithobius forficatus
0.1 substitutions/site
Strongylidae
Cloacinidae
Chabertiidae
Strongylidae
Cloacinidae
Ancylostomatidae
Filaroididae
Angiostrongylidae
Metastrongylidae
Protostrongylidae
Dictyocaulidae
Syngamidae
Haemonchidae
Trichostrongylidae
Cooperiidae
Heligmonellidae
Molineidae
Heligmosomatidae
Rhabditidae
Neodiplogasteridae
Heterorhabditidae
Aphelenchoididae
Aphelenchidae
Panagrolaimidae
Strongyloididae
Alloionematidae
Steinernematidae
Ascarididae
Toxocaridae
Anisakidae
Ascarididae
Gnathostomatidae
Cucullanidae
Heterakidae
Ascaridiidae
Rhigonematidae
Dracunculidae
Philometridae
Camallanidae
Onchocercidae
Setariidae
Gongylonematidae
Thelaziidae
Physalopteridae
Oxyuridae
Heteroxynematidae
Oxyuridae
Meloidogynidae
Pratylenchidae
Heteroderidae
Pratylenchidae
Cephalobidae
Plectidae
Rhabditomorpha
Diplogasteromorpha
Rhabditomorpha
Tylenchomorpha (Aphelenchoidea)
Panagrolaimomorpha
Ascaridomorpha
Gnathostomatomorpha
Ascaridomorpha
Rhigonematomorpha
Dracunculoidea
Spiruromorpha
Oxyuridomorpha
Tylenchomorpha (Tylenchoidea)
Cephalobomorpha
Plectida
Mermithida
Dorylaimida
Trichinellida
Arthropod outgroups
Rhabditina
Tylenchina
Spirurina
Tylenchina
Chromadorea
Enoplea
