# Supplementary material for: The mitochondrial genome of Acrobeloides varius (Cephalobomorpha) confirms non-monophyly of Tylenchina (Nematoda)
Source: PeerJ. 2020 May 13;8:e9108. doi: 10.7717/peerj.9108 (PMC7229770; doi:10.7717/peerj.9108)
Supplement: Figure S7 — Bayesian posterior probabilities (BPP) were estimated after discarding the initial 250 trees (the first 25 × 104 generations) as burn-in. BPP values <0.7 are not shown. [file peerj-08-9108-s011.pdf]

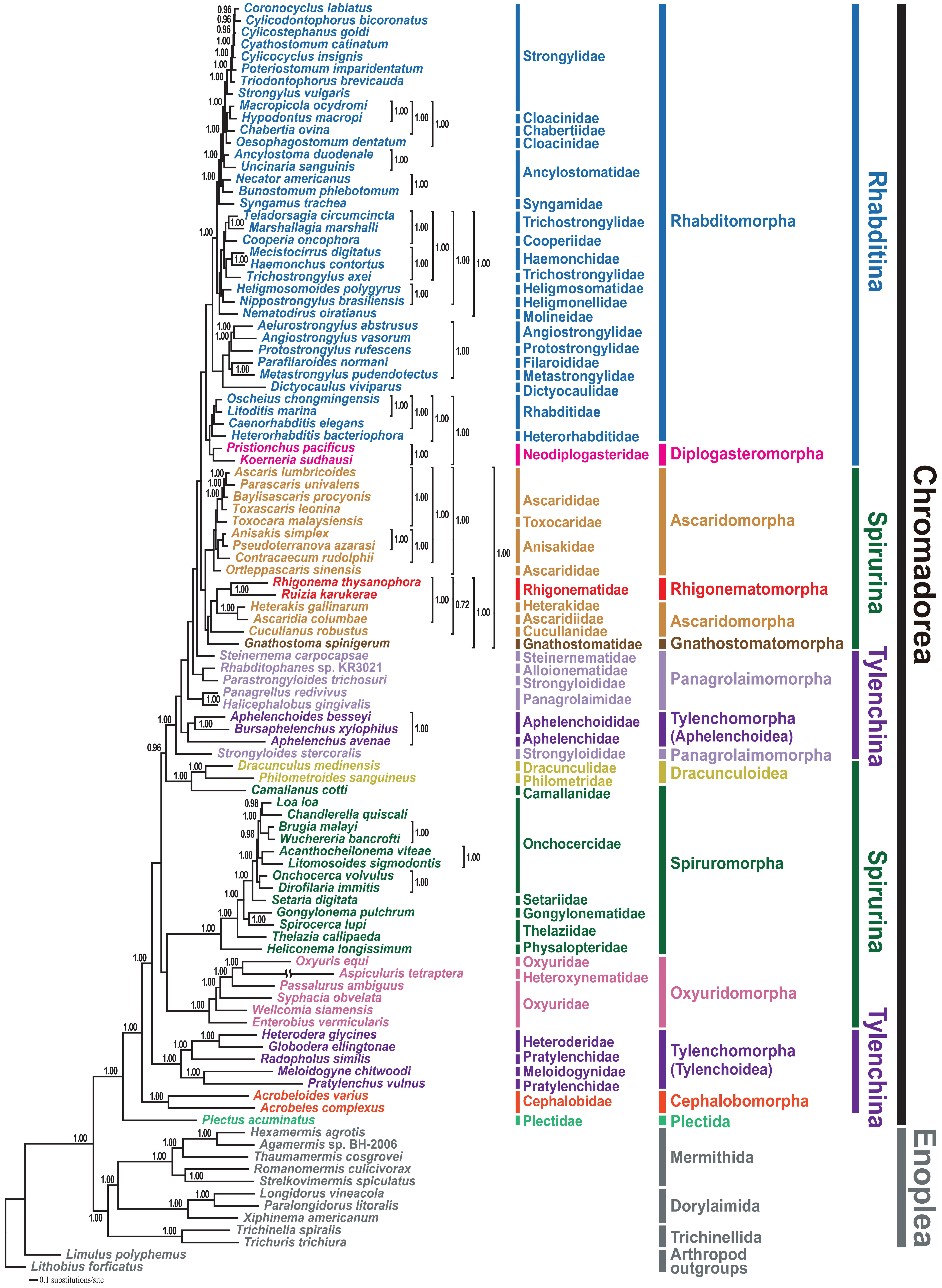
Coronocyclus labiatus
Cylicodontophorus bicoronatus
Cylicostephanus goldi
Cyathostomum catinatum
Cylicocyclus insignis
Poteriostomum imparidentatum
Triodontophorus brevicauda
Strongylus vulgaris
Macropicola ocydromi
Hypodontus macropi
Chabertia ovina
Oesophagostomum dentatum
Ancylostoma duodenale
Uncinaria sanguinis
Necator americanus
Bunostomum phlebotomum
Syngamus trachea
Teladorsagia circumcincta
Marshallagia marshalli
Cooperia oncophora
Mecistocirrus digitatus
Haemonchus contortus
Trichostrongylus axei
Heligmosomoides polygyrus
Nippostrongylus brasiliensis
Nematodirus oiratianus
Aelurostrongylus abstrusus
Angiostrongylus vasorum
Protostrongylus rufescens
Parafilaroides normani
Metastrongylus pudendotectus
Dictyocaulus viviparus
Oscheius chongmingensis
Litoditis marina
Caenorhabditis elegans
Heterorhabditis bacteriophora
Pristionchus pacificus
Koerneria sudhausi
Ascaris lumbricoides
Parascaris univalens
Baylisascaris procyonis
Toxascaris leonina
Toxocara malaysiensis
Anisakis simplex
Pseudoterranova azarasi
Contracaecum rudolphii
Ortleppascaris sinensis
Rhigonema thysanophora
Ruizia karukerae
Heterakis gallinarum
Ascaridia columbae
Cucullanus robustus
Gnathostoma spinigerum
Steinernema carpocapsae
Rhabditophanes sp. KR3021
Parastrongyloides trichosuri
Panagrellus redivivus
Halicephalobus gingivalis
Aphelenchoides besseyi
Bursaphelenchus xylophilus
Aphelenchus avenae
Strongyloides stercoralis
Dracunculus medinensis
Philometroides sanguineus
Camallanus cotti
Loa loa
Chandlerella quiscali
Brugia malayi
Wuchereria bancrofti
Acanthocheilonema viteae
Litomosoides sigmodontis
Onchocerca volvulus
Dirofilaria immitis
Setaria digitata
Gongylonema pulchrum
Spirocerca lupi
Thelazia callipaeda
Heliconema longissimum
Oxyuris equi
Aspiculuris tetraptera
Passalurus ambiguus
Syphacia obvelata
Wellcomia siamensis
Enterobius vermicularis
Heterodera glycines
Globodera ellingtonae
Radopholus similis
Meloidogyne chitwoodi
Pratylenchus vulnus
Acrobeloides varius
Acrobeles complexus
Plectus acuminatus
Hexamermis agrotis
Agamermis sp. BH-2006
Thaumamermis cosgrovei
Romanomermis culicivorax
Strelkovimermis spiculatus
Longidorus vineacola
Paralongidorus litoralis
Xiphinema americanum
Trichinella spiralis
Trichuris trichiura
Limulus polyphemus
Lithobius forficatus
0.1 substitutions/site
Strongylidae
Cloacinidae
Chabertiidae
Cloacinidae
Ancylostomatidae
Syngamidae
Trichostrongylidae
Cooperiidae
Haemonchidae
Trichostrongylidae
Heligmosomatidae
Heligmonellidae
Molineidae
Angiostrongylidae
Protostrongylidae
Filaroididae
Metastrongylidae
Dictyocaulidae
Rhabditidae
Heterorhabditidae
Neodiplogasteridae
Ascarididae
Toxocaridae
Anisakidae
Ascarididae
Rhigonematidae
Heterakidae
Ascaridiidae
Cucullanidae
Gnathostomatidae
Steinernematidae
Alloionematidae
Strongyloididae
Panagrolaimidae
Aphelenchoididae
Aphelenchidae
Strongyloididae
Dracunculidae
Philometridae
Camallanidae
Onchocercidae
Setariidae
Gongylonematidae
Thelaziidae
Physalopteridae
Oxyuridae
Heteroxynematidae
Oxyuridae
Heteroderidae
Pratylenchidae
Meloidogynidae
Pratylenchidae
Cephalobidae
Plectidae
Rhabditomorpha
Diplogasteromorpha
Ascaridomorpha
Rhigonematomorpha
Ascaridomorpha
Gnathostomatomorpha
Panagrolaimomorpha
Tylenchomorpha (Aphelenchoidea)
Panagrolaimomorpha
Dracunculoidea
Spiruromorpha
Oxyuridomorpha
Tylenchomorpha (Tylenchoidea)
Cephalobomorpha
Plectida
Mermithida
Dorylaimida
Trichinellida
Arthropod outgroups
Rhabditina
Spirurina
Tylenchina
Spirurina
Tylenchina
Chromadorea
Enoplea
