# Supplementary material for: The mitochondrial genome of Acrobeloides varius (Cephalobomorpha) confirms non-monophyly of Tylenchina (Nematoda)
Source: PeerJ. 2020 May 13;8:e9108. doi: 10.7717/peerj.9108 (PMC7229770; doi:10.7717/peerj.9108)
Supplement: Figure S8 — Bayesian posterior probabilities (BPP) were estimated after discarding the initial 250 trees (the first 25 × 104 generations) as burn-in. BPP values <0.7 are not shown. [file peerj-08-9108-s012.pdf]

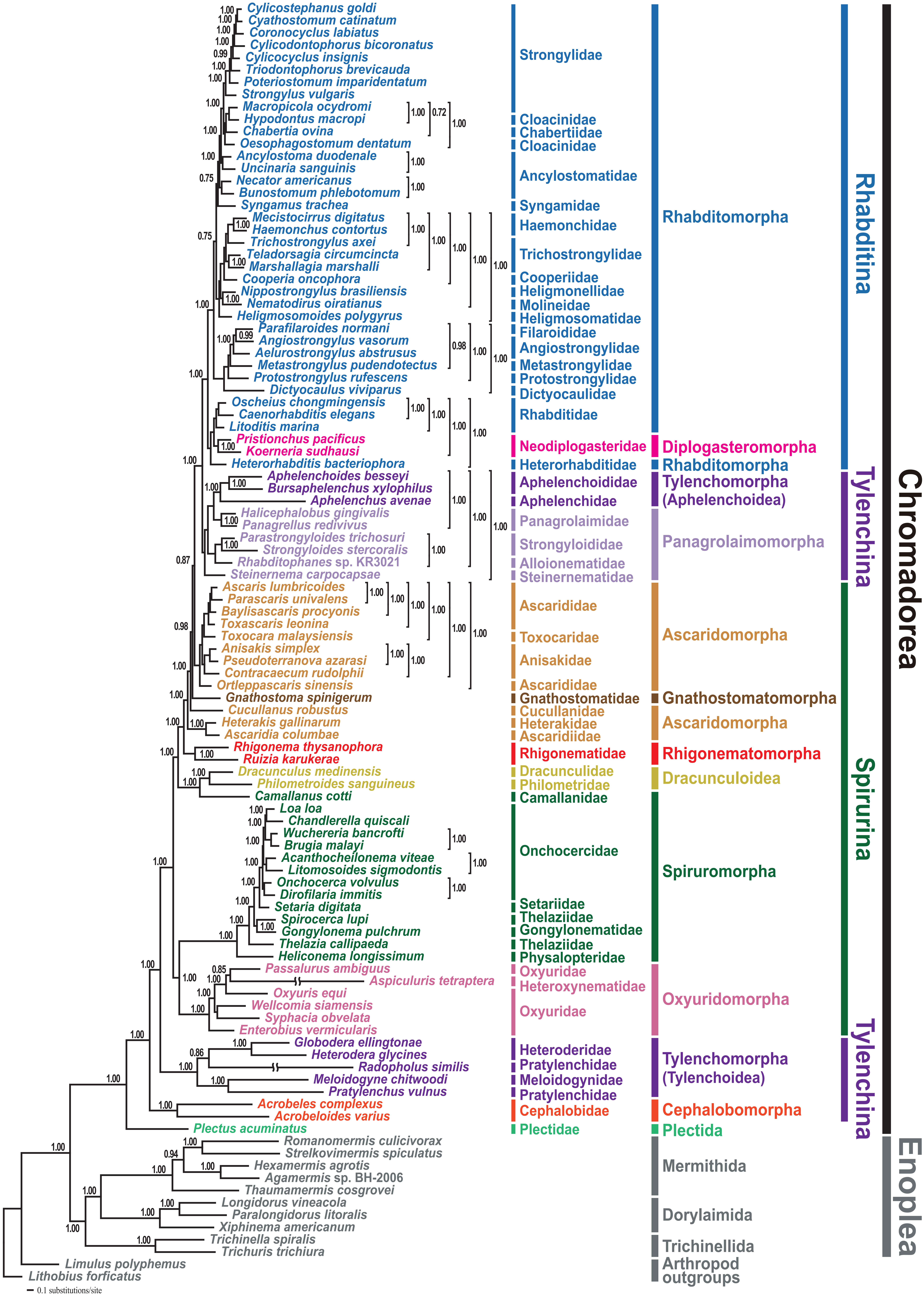

Cylicostephanus goldi
Cyathostomum catinatum
Coronocyclus labiatus
Cylicodontophorus bicoronatus
Cylicocyclus insignis
Triodontophorus brevicauda
Poteriostomum imparidentatum
Strongylus vulgaris
Macropicola ocydromi
Hypodontus macropi
Chabertia ovina
Oesophagostomum dentatum
Ancylostoma duodenale
Uncinaria sanguinis
Necator americanus
Bunostomum phlebotomum
Syngamus trachea
Mecistocirrus digitatus
Haemonchus contortus
Trichostrongylus axei
Teladorsagia circumcincta
Marshallagia marshalli
Cooperia oncophora
Nippostrongylus brasiliensis
Nematodirus oiratianus
Heligmosomoides polygyrus
Parafilaroides normani
Angiostrongylus vasorum
Aelurostrongylus abstrusus
Metastrongylus pudendotectus
Protostrongylus rufescens
Dictyocaulus viviparus
Oscheius chongmingensis
Caenorhabditis elegans
Litoditis marina
Pristionchus pacificus
Koerneria sudhausi
Heterorhabditis bacteriophora
Aphelenchoides besseyi
Bursaphelenchus xylophilus
Aphelenchus avenae
Halicephalobus gingivalis
Panagrellus redivivus
Parastrongyloides trichosuri
Strongyloides stercoralis
Rhabditophanes sp. KR3021
Steinernema carpocapsae
Ascaris lumbricoides
Parascaris univalens
Baylisascaris procyonis
Toxascaris leonina
Toxocara malaysiensis
Anisakis simplex
Pseudoterranova azarasi
Contracaecum rudolphii
Ortleppascaris sinensis
Gnathostoma spinigerum
Cucullanus robustus
Heterakis gallinarum
Ascaridia columbae
Rhigonema thysanophora
Ruizia karukerae
Dracunculus medinensis
Philometroides sanguineus
Camallanus cotti
Loa loa
Chandlerella quiscali
Wuchereria bancrofti
Brugia malayi
Acanthocheilonema viteae
Litomosoides sigmodontis
Onchocerca volvulus
Dirofilaria immitis
Setaria digitata
Spirocerca lupi
Gongylonema pulchrum
Thelazia callipaeda
Heliconema longissimum
Passalurus ambiguus
Aspiculuris tetraptera
Oxyuris equi
Wellcomia siamensis
Syphacia obvelata
Enterobius vermicularis
Globodera ellingtonae
Heterodera glycines
Radopholus similis
Meloidogyne chitwoodi
Pratylenchus vulnus
Acrobeles complexus
Acrobeloides varius
Plectus acuminatus
Romanomermis culicivorax
Strelkovimermis spiculatus
Hexamermis agrotis
Agamermis sp. BH-2006
Thaumamermis cosgrovei
Longidorus vineacola
Paralongidorus litoralis
Xiphinema americanum
Trichinella spiralis
Trichuris trichiura
Limulus polyphemus
Lithobius forficatus
0.1 substitutions/site
Strongylidae
Cloacinidae
Chabertiidae
Cloacinidae
Ancylostomatidae
Syngamidae
Haemonchidae
Trichostrongylidae
Cooperiidae
Heligmonellidae
Molineidae
Heligmosomatidae
Filaroididae
Angiostrongylidae
Metastrongylidae
Protostrongylidae
Dictyocaulidae
Rhabditidae
Neodiplogasteridae
Heterorhabditidae
Aphelenchoididae
Aphelenchidae
Panagrolaimidae
Strongyloididae
Alloionematidae
Steinernematidae
Ascarididae
Toxocaridae
Anisakidae
Ascarididae
Gnathostomatidae
Cucullanidae
Heterakidae
Ascaridiidae
Rhigonematidae
Dracunculidae
Philometridae
Camallanidae
Onchocercidae
Setariidae
Thelaziidae
Gongylonematidae
Thelaziidae
Physalopteridae
Oxyuridae
Heteroxynematidae
Oxyuridae
Heteroderidae
Pratylenchidae
Meloidogynidae
Pratylenchidae
Cephalobidae
Plectidae
Rhabditomorpha
Diplogasteromorpha
Rhabditomorpha
Tylenchomorpha (Aphelenchoidea)
Panagrolaimomorpha
Ascaridomorpha
Gnathostomatomorpha
Ascaridomorpha
Rhigonematomorpha
Dracunculoidea
Spiruromorpha
Oxyuridomorpha
Tylenchomorpha (Tylenchoidea)
Cephalobomorpha
Plectida
Mermithida
Dorylaimida
Trichinellida
Arthropod outgroups
Rhabditina
Tylenchina
Spirurina
Tylenchina
Chromadorea
Enoplea
